# 13CFLUX2—high-performance software suite for ^13^C-metabolic flux analysis

**DOI:** 10.1093/bioinformatics/bts646

**Published:** 2012-10-30

**Authors:** Michael Weitzel, Katharina Nöh, Tolga Dalman, Sebastian Niedenführ, Birgit Stute, Wolfgang Wiechert

**Affiliations:** ^1^Institute of Bio- and Geosciences, IBG-1: Biotechnology and ^2^JARA High Performance Computing, Forschungszentrum Jülich GmbH, 52428 Jülich, Germany

## Abstract

**Summary:** ^13^C-based metabolic flux analysis (^13^C-MFA) is the state-of-the-art method to quantitatively determine *in vivo* metabolic reaction rates in microorganisms. 13CFLUX2 contains all tools for composing flexible computational ^13^C-MFA workflows to design and evaluate carbon labeling experiments. A specially developed XML language, FluxML, highly efficient data structures and simulation algorithms achieve a maximum of performance and effectiveness. Support of multicore CPUs, as well as compute clusters, enables scalable investigations. 13CFLUX2 outperforms existing tools in terms of universality, flexibility and built-in features. Therewith, 13CFLUX2 paves the way for next-generation high-resolution ^13^C-MFA applications on the large scale.

**Availability and implementation:** 13CFLUX2 is implemented in C++ (ISO/IEC 14882 standard) with Java and Python add-ons to run under Linux/Unix. A demo version and binaries are available at www.13cflux.net.

**Contact:** info@13cflux.net or k.noeh@fz-juelich.de

**Supplementary information:** Supplementary data are available at *Bioinformatics* online.

## 1 MOTIVATION

Metabolic flux analysis with carbon labeling experiments (^13^C-MFA) matured as the state-of-the-art technique to infer directly immeasurable *in vivo* central metabolic reaction rates, the *fluxome*, by rigorous mathematical modeling (Sauer, 2006; Wiechert, 2001). Progress in measurement techniques and scaled-down experimentation has raised the experimental throughput and coverage to which isotope-labeled tracers in the metabolism are quantified (Fan and Lane, 2008). This has encouraged the usage of ^13^C-MFA for cell-wide analyses of complex cells such as eukaryotes, mammalian cells or fungi (Zamboni, 2011). Such applications drastically increase the computational burden and cannot be adequately treated with existing all-purpose software.

Built on experiences made with its successful predecessor 13CFLUX, the high-performance software suite 13CFLUX2 is designed to overcome computational and modeling limitations to increase the flexibility and scope of ^13^C-MFA. Major unique features of 13CFLUX2 are (i) tailor-made algorithms in combination with a novel code generation approach leading to highly efficient machine code, (ii) the XML-based document format FluxML to specify ultimate universal models and all kind of measurements, (iii) support of high-performance computing environments, and (iv) seamless setup of user-defined processing pipelines for serial evaluations. Moreover, the multi-platform software Omix may be used for convenient modeling and visualization purposes (Droste *et al.*, 2011). With respect to these features, 13CFLUX2 exceeds the functionality of existing ^13^C-MFA software systems, namely, Metran and FiatFlux, as well as the 13CFLUX clones OpenFlux, C13, FIA, NMR2FLUX and influx_s (Cvijovic *et al.*, 2010; Quek *et al.*, 2009; Sokol *et al.*, 2012; Sriram *et al.*, 2004; Srour *et al.*, 2011; Yoo *et al.*, 2008; Zamboni *et al.*, 2005).

## 2 METHODS AND IMPLEMENTATION

**Fig. 1.**
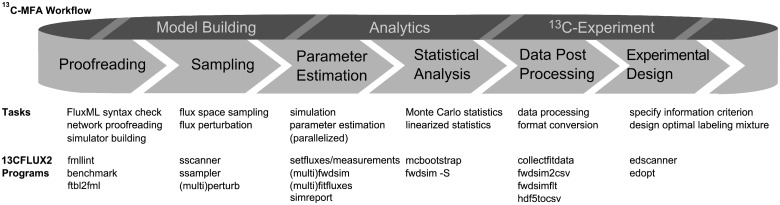
Overview scheme of typical steps within the ^13^C-MFA workflow and related 13CFLUX2 tools (for additional details see Supplementary Material)

13CFLUX2 is implemented in C++ and consists of 130 000+ lines of strictly object-oriented, portable and validated ISO/ANSI C++ code running on Linux/Unix platforms. The modular software suite comprises 21 modules, which make up the core components of ^13^C-MFA research workflows (see Fig. 1). 13CFLUX2 is equipped with a comprehensive error handling architecture, while built-in automatic debugging, logging, assertions and stack traces do not affect the performance of the production-level code. Several additional Java/Perl/Python-based programs ease parsing of analysis results or performing post-processing tasks.

### 2.1 FluxML document format

For the specification of metabolic and isotopic reaction networks, the XML-based document format FluxML has been developed. Semantically similar to SBML, FluxML contains substantial extentions for representing ^13^C-MFA specific concepts, i.e. the modeling of atom mappings (an example FluxML file is available as Supplementary Material). Special focus has been laid on the formulation of universal stoichiometric constraints, as well as flux and labeling measurements that both can be specified in a textual or Content-MathML notation (www.w3.org/math). Besides build-in support for MS(/MS)- and ^1^H/^13^C-NMR-type measurements by convenient short notations, specification of generic measurements is possible. More than 400 syntactical and semantical errors are detected and indicated by expressive error/warning messages.

### 2.2 HPC algorithms for ultimate performance

Simulating the cells’ isotopic labeling state is the performance-critical core procedure of ^13^C-MFA workflows. Cumomer- and EMU-based approaches are numerically stable as they inhere a (quasi-) linear model structure (Antoniewicz *et al.*, 2007; Wiechert *et al.*, 1999). In 13CFLUX2, an interpreter-based network generator assembles both, the Cumomer and EMU equations from the FluxML-based network specification. New algorithms for an on-the-fly in-depth dependency analysis of the emerging systems enable an optimal network reduction resulting in systems of minimal size. Advanced graph decomposition and path tracing algorithms exploit characteristic connectivity properties of the Cumomer/EMU networks, like immanent sparsity and isomorphism (Weitzel *et al.*, 2007). The resulting reduced labeling systems are translated into a cascade of symbolic equation systems, allowing for a highly efficient numerical solution, or alternatively, exact solutions based on arbitrary precision arithmetic. Optionally, the symbolic equation systems can be compiled into efficient machine code. Notably, the generation of analytical solutions is possible for large-scale network models with almost linear run time with respect to the number of labeled species. Gradients for statistical analyses and optimizers are derived with maximum numerical precision based on symbolic differentiation. Sharing the same mathematical structure with the original (reduced) systems, their numerical solution, is likewise efficiently performed. Exact derivatives are provided optionally.

Code performance is demonstrated with an *Escherichia coli* network slightly adapted from (Weitzel *et al.*, 2007) containing 197 metabolites and 292 reactions. *S*-adenosyl-l-methionine (15 carbons) contributes to almost 65% to the total 75 549 labeled species. For a typical GC/MS-type measurement setup, Cumomer-based simulation takes 10.8 ms, whereas for the EMU variant, 2.73 ms are measured on a 2.93 GHz XEON machine with 4 MB L2 cache running Linux 2.6. On average, we found 13CFLUX2 to be 100 – 10 000 times faster compared with 13CFLUX.

## 3 FLUX ANALYSIS WORKFLOW(S) WITH 13CFLUX2

Figure 1 surveys the main tasks within ^13^C-MFA workflows. All required ingredients including the metabolic and isotopic network, the stoichiometric constraints, input species and the measurement configuration are formulated in the model’s FluxML document. Subsequent to the proofreading step, the FluxML document is validated (*fmllint*). A feasible basis of the stoichiometric null space is determined with regard to the modeler’s selection. Constraint-compliant initial values for the free fluxes are generated by state-of-the-art samplers (*sscanner*, *ssampler*). Sensitivity and identifiability analyses allow detecting non-identifiable fluxes to avoid flawed parameter estimation artifacts (*fwdsim –S*, *multi-fwdsim*). Calculation of flux maps and their statistical quality assessment (*multi-fitfluxes, mcbootstrap*) relies on the powerful optimization libraries IPOPT (www.coin-or.org/ipopt) and NAG C (www.nag.co.uk). On top of the workflow, the experimental design programs *edscanner* and *edopt* determine most informative input labeling species based on D-/A-/E-/M-information measures (Atkinson and Donev, 1992).

All 13CFLUX2 modules support standardized *stdin*/*stout* operations enabling seamless composition of tailor-made scalable processing workflows, e.g. by using scripting languages or web service wrappers. For data post-processing, simulation results are exported to HDF5/CSV formats. Resulting flux maps can be readily visualized in the software Omix. To assist rapid application development, the symbolic equation systems can be exported as MathML documents (e.g. for computer algebra systems) and as MATLAB^™^-based fully functional labeling simulator.

## 4 CONCLUSIONS

^13^C-MFA reliably quantifies *in vivo* activities of cellular carbon redistribution. The next-generation software 13CFLUX2 addresses the challenges posed by upcoming large-scale and high-throughput applications. Therewith, 13CFLUX2 shifts paradigms of ^13^C-MFA toward semi-supervised large-scale high-resolution applications. In combination with the graphical tool Omix, 13CFLUX2 is a software suite for both computational scientists and researchers from life science.

## Supplementary Material

Supplementary Data
