# Supplementary material for: 13CFLUX2—high-performance software suite for 13C-metabolic flux analysis
Source: Bioinformatics. 2012 Oct 30;29(1):143–5. doi: 10.1093/bioinformatics/bts646 (PMC3530911; doi:10.1093/bioinformatics/bts646)
Supplement: Supplementary Data [file supp_29_1_143__index.html]

13CFLUX2 – High-Performance Software Suite for 13C-Metabolic Flux Analysis — 13CFLUX2—high-performance software suite for 13C-metabolic flux analysis — Supplementary Data 

# 13CFLUX2—high-performance software suite for 13C-metabolic flux analysis

## Supplementary Data

files

**Files in this Data Supplement:**

- Supplementary Data - pdf file
- Supplementary Data - pdf file
