# Supplementary material for: 13CFLUX2—high-performance software suite for 13C-metabolic flux analysis
Source: Bioinformatics. 2012 Oct 30;29(1):143–5. doi: 10.1093/bioinformatics/bts646 (PMC3530911; doi:10.1093/bioinformatics/bts646)
Supplement: Supplementary Data [file supp_bts646_Weitzel_et_al_2012-Application_Note_13CFLUX2-BIOINF-2012-1344_resubmission_Supp1-13CFLUX2_FACTSHEET.pdf]

Modeling and Simulation Group
IBG-1: Biotechnology
Forschungszentrum Jülich GmbH
52425 Jülich, Germany
{info,k.noeh}@fz-juelich.de

The 13CFLUX2 Fact Sheet

- **A complete modeling and data evaluation environment for ^{13}C-metabolic flux analysis (^{13}C-MFA)**
  - Second generation software *13CFLUX2* bases on new ideas: it does not share a single line of code with old *13CFLUX*
  - Provides highly efficient implementations of the Cumomer and EMU simulation algorithms
  - Flexible description of any measurement specification
  - High performance simulation of isotope labeling experiments
  - A priori (optimal) experimental design for isotope labeling experiments
  - Flux estimation with subsequent detailed statistical analysis
  - Graphical network editor *Omix* as modeling front-end for editing of metabolic and isotope transition networks as well as measurement specifications (http://www.13cflux.net/omix)
  - Visualization of results directly on the network drawing using *Omix* or *MATLAB ™*
- **High performance algorithms for high performance ^{13}C-MFA applications**
  - Flux analysis for large-scale metabolic network models
  - For typical metabolic network models 10^2 to 10^4 times faster than *13CFLUX*
  - Optimal choice of simulation method (Cumomer, EMU) depending on measurement specifications
  - Interpreter-based network generator provides fast startup times
  - Topological analysis of network graphs and optimal network reduction
  - Linearized and non-linear statistical analyses
  - Improved numerical precision (especially for larger networks)
  - Support for SMP machines and cluster architectures (parallel parameter fitting, Monte Carlo Bootstrap)
- **FluxML (XML) documents for metabolic and isotope network specification**
  - Extensible and more flexible than old FTBL file format
  - Powerful conversion tool FTBL2FluxML to support older *13CFLUX* models
  - Free-form constraint equations using MathML or textual notation
  - Built-in support for MS, MS/MS, ^{1}H-NMR, ^{13}C-NMR measurements
  - Support for arbitrary measurement equations using MathML or textual notation
  - Support for various constraint/ measurement/ input mixture configurations in one document
- **Arbitrary precision by symbolic and algebraic methods**
  - Symbolic handling and analysis of stoichiometric constraints
  - Export of stoichiometric equations
  - Exact solutions (rational number arithmetic) and symbolic solutions
  - Exact derivatives for faster convergence of gradient-based optimization algorithms
  - Exact parameter sensitivities based on symbolic differentiation
  - Useful for studying numerical error propagation/ analysis of numerical problems
- **Advanced optimization toolbox providing different optimization algorithms**
  - Advanced sampler for feasible flux initialization
  - SQP-, NLP-based optimization
  - *Ipopt* as primary optimizer (http://projects.coin-or.org/Ipopt)
  - Easily adaptable to commercially available optimization library NAG-C (http://www.nag.co.uk)
- **Interfaces and Visualization**
  - Data exchange between applications is established using XML and HDF5 documents
  - All applications support *stdin/ stdout operation* (i.e. applications act as *filters*)
  - Well-suited for cluster computing, e.g. by using (MPI-based) wrappers
  - Applications export numerical data as HDF5 files (→ *MATLAB ™*) and CSV (→ Spreadsheet)
  - FluxML can be imported/ exported by the graphical network editor *Omix*
  - Symbolic and numerical data can be imported, post-processed and visualized in *MATLAB ™*
  - A fully functional, tailor-made simulator can be exported as *MATLAB ™* function
- **Tidy and robust C++/ Python codebase**
  - Consists of 130.000+ lines of portable and validated ISO/ ANSI C++
  - Compilable on state-of-the-art Linux and Unix platforms (tested for Ubuntu, Debian, OpenSuSE, Fedora)
  - Comprehensive handling of errors and exceptions, not affecting performance of the production code: built-in automatic debugging, logging, assertions and stack traces
  - Revision control/ SCM based on *Subversion* (http://subversion.apache.org)
  - Build environment based on the *GNU build system (autoconf, automake)*
