# Supplementary material for: 13CFLUX2—high-performance software suite for 13C-metabolic flux analysis
Source: Bioinformatics. 2012 Oct 30;29(1):143–5. doi: 10.1093/bioinformatics/bts646 (PMC3530911; doi:10.1093/bioinformatics/bts646)
Supplement: Supplementary Data [file supp_bts646_Weitzel_et_al_2012-Application_Note_13CFLUX2-BIOINF-2012-1344_resubmission_Supp2-13CFLUX2_REFERENCE_MANUAL.pdf]

13CFLUX2 Reference Manual

Reference Documentation for the software suite 13CFLUX2
Version 1.0 – October 2012

Michael Weitzel, Tolga Dalman, Katharina Nöh
IBG-1: Biotechnology, Research Centre Julich (Forschungszentrum Jülich GmbH), Jülich, Germany

This text is a collection of man-pages for the software suite 13CFLUX2 (release October 2012)

Copyright © 2009-2012 IBG-1: Biotechnology, Forschungszentrum Jülich GmbH

Permission is granted to copy and distribute verbatim copies of this document under the terms of the 13CFLUX2 license agreement. A copy of the license is included in the section entitled

"13CFLUX2 license"

Published by the Modeling and Simulation Group
IBG-1: Biotechnology
Forschungszentrum Jülich GmbH
52428 Jülich
Germany
info@13cflux.net
www.13cflux.net

Contents

13CFLUX2 programs

benchmark 4

collectfitdata 6

edopt 8

edscanner 13

fitfluxes 17

fmllint 21

ftbl2fml 23

fwdsim 26

fwdsim2csv 31

fwdsimflt 32

hdf5tocsv 34

mcbootstrap 35

multifit 37

multifitfluxes 39

multifwdsim 43

multiperturb 46

perturb 48

setfluxes 50

setmeasurements 52

simreport 54

sscanner 55

ssampler 58

Appendix

13CFLUX2 – a short reference guide 62

13CFLUX2 license 72

NAME

benchmark − performance benchmark for the 13CFLUX 2 software

SYNOPSIS

benchmark [*options*]

DESCRIPTION

The program **benchmark** can be used to generate a performance benchmark for different linear solvers. The tool gives useful information about the simulated cumomer / EMU network and performs an analysis of the computational graphs. For details the reader is referred to Weitzel et al. BMC Bioinformatics 8:315, 2007.

COMMON OPTIONS

−h, −−help
Show a brief help for all command line options.

−i, −−in <FILE> [default: stdin]
The name of the FluxML (XML) input file. If omitted, the FluxML document is expected on standard input.

−s, −−solvers X,Y,... [default: all built−in solvers]
This option takes a comma−separated list of names of the linear solvers to be used for the performance benchmark.

−L, −−list−solvers
This option causes a list of valid solver names to be generated.

−S, −−statistics
This option causes additional statistics on the (reduced) network and its computational graphs to be generated.

−c, −−configure <CFG> [default: 'default']
Because FluxML documents may contain several **<configuration/>** elements this option allows to specify the configuration that should be used for the simulation. If this option is omitted it is assumed that the FluxML document contains a configuration with the name "default".

−l, −−log DEST
Specify the destination for the internal logging. In the most simple case **DEST** is a file name of a log file. In case the file exists new log messages are appended. Apart from log files it is possible to publish log messages to file descriptors, UNIX domain sockets, UDP and SCTP ports, and a small graphical user interface.

A file descriptor is specified by **fd:[num]**, where **[num]** is the number of the file descriptor.

A unix domain socket in the local file system is specified by **unix:[name]**, where **[name]** is the name of the socket file

A UDP or (connectionless) SCTP port is specified by **[proto]:[host]:[port]**, where **[proto]** is either "udp" or "sctp" and **[host]** is the name of the destination host and **[port]** is a UDP or SCTP port number on the destination host. Please note that the length of log messages is bounded by the minimum safe UDP packet size − log messages containing more than 548 characters will be truncated.

Finally, log messages can also be sent to a small GUI by specifying the destination **@gui@**. The GUI requires a working Perl/Tk installation and a running X server.

In order to capture all log messages concerning the command line processing this option should be specified in front of all other options.

−v, −−verbose 0..10 [default: 5]
Specify the verbosity 0, 1, ..., 10 of generated / emitted log messages. The meaning of the different log

levels is as follows:

- **0 (QUIET)** do not emit log messages at all.
- **1 (ERROR)** only emit severe error messages.
- **2 (WARNING)** only report severe errors and warnings.
- **3 (NOTICE)** report all errors and warnings including important informal messages.
- **4 (INFO)** report all errors, warnings and all informal messages.
- **5 (THROW)** in case of an exception, try to give a diagnosis of the error; sometimes even gives a backtrace of the current function stack.
- **6 (DEBUG0)** emit the more important debugging messages.
- **7 (DEBUG1)** emit the less important debugging messages
- **8 (DEBUG2)** emit the superfluous debugging messages
- **9 (DEBUG3)** emit annoying debugging messages.
- **10 (DEBUG4)** don't dare to use it!

EXAMPLES

Perform 123 benchmark runs using only solver "CSparseLU":

benchmark −r 123 −s CSparseLU −i network.fml

SEE ALSO

fwdsim(1), fitfluxes(1)

AUTHOR

Michael Weitzel

This manpage was written by Michael Weitzel <mich@el−weitzel.de>.

NAME

collectfitdata − collect together simulation results into a HDF5 file.

SYNOPSIS

collectfitdata [*options*] [−o <FILE>]

DESCRIPTION

Collectfitdata is a tool for collecting together the results (i.e. net fluxes, exchange fluxes, and measurement values) from multiple FWDSIM (XML) files (see **fwdsim**(5)). The collected data is saved to a HDF5 file.

COMMON OPTIONS

−h, −−help

Show a brief help for all command line options.

−f, −−filelist <PATH>

Path to a directory containing FWDSIM files. It is assumed, that these FWDSIM files are created by fitfluxes or fwdsim from the same FluxML file! If omitted, collectfitdata accepts a single FWDSIM file from stdin.

−o, −−out <FILE>

The name of the HDF5 output file. This option must be supplied (HDF5 files are never written to standard output).

−a, −−append

When specified, this option causes the HDF5 file to be appended, if the file existed before. Otherwise a new HDF5 is created.

−l, −−log DEST

Specify the destination for the internal logging. In the most simple case **DEST** is a file name of a log file. In case the file exists new log messages are appended. Apart from log files it is possible to publish log messages to file descriptors, UNIX domain sockets, UDP and SCTP ports, and a small graphical user interface.

A file descriptor is specified by **fd:[num]**, where **[num]** is the number of the file descriptor.

A unix domain socket in the local file system is specified by **unix:[name]**, where **[name]** is the name of the socket file

A UDP or (connectionless) SCTP port is specified by **[proto]:[host]:[port]**, where **[proto]** is either "udp" or "sctp" and **[host]** is the name of the destination host and **[port]** is a UDP or SCTP port number on the destination host. Please note that the length of log messages is bounded by the minimum safe UDP packet size − log messages containing more than 548 characters will be truncated.

Finally, log messages can also be sent to a small GUI by specifying the destination **@gui@**. The GUI requires a working Perl/Tk installation and a running X server.

In order to capture all log messages concerning the command line processing this option should be specified in front of all other options.

−v, −−verbose 0..10 [default: 5]

Specify the verbosity 0, 1, ..., 10 of generated / emitted log messages. The meaning of the different log levels is as follows:

- **0 (QUIET)** do not emit log messages at all.
- **1 (ERROR)** only emit severe error messages.
- **2 (WARNING)** only report severe errors and warnings.
- **3 (NOTICE)** report all errors and warnings including important informal messages.

- **4 (INFO)** report all errors, warnings and all informal messages.
- **5 (THROW)** in case of an exception, try to give a diagnosis of the error; sometimes even gives a backtrace of the current function stack.
- **6 (DEBUG0)** emit the more important debugging messages.
- **7 (DEBUG1)** emit the less important debugging messages
- **8 (DEBUG2)** emit the superfluous debugging messages
- **9 (DEBUG3)** emit annoying debugging messages.
- **10 (DEBUG4)** don't dare to use it!

CONTENT CONTROL OPTIONS

−m, −−measurements
With this option, measurement entries are also collected into the HDF5 file. Specifically, measurement group values from the FWDSIM file under the node <measurements> are written into the HDF5 file into the field /measurements.

−F, −−free−only
Only collect free variables into the HDF5 file. Constrained and dependent fluxes are omitted.

−N, −−omit−net−fluxes
With this option defined, net fluxes are not collected into the HDF5 file.

−X, −−omit−xch−fluxes
With this option defined, xch fluxes are not collected into the HDF5 file.

EXAMPLES

Collecting together the results from a directory containing a number of FWDSIM (XML) files can be performed like this:

collectfitdata −f fwdsim_dir −o results.hdf5

Single FWDSIM (XML) files can be piped into collectfitdata:

cat data.fwd | collectfitdata −o results.hdf5

Collecting only free net fluxes is performed as follows:

collectfitdata −f fwdsim_dir −o results.hdf5 −X −F

SEE ALSO

fwdsim(1), fitfluxes(1), multifit(1)

AUTHOR

Tolga Dalman

This manpage was written by Tolga Dalman <info@13cflux.net> and Michael Weitzel <mich@el−weitzel.de>.

NAME

edopt − substrate mixture optimization (experimental design).

SYNOPSIS

edopt [*options*]

DESCRIPTION

Given a FluxML file with an optimized flux distribution the program **edopt** can be used to generate an optimal design for a subsequent isotope labeling experiment.

For this purpose, **edopt** performs a linearized statistical analysis and determines the mixture of substrates which minimizes the volume of the flux distribution's covariance ellipsoid, i.e. allows the most accurate flux determination having the smallest standard deviations.

Be aware that successful experimental design may require to specify an error model the extrapolation of standard errors of simulated measurements. Details can be found in **fluxml**(5) (element "errormodel").

OPTIONS

−h, −−help
Show a brief help for all command line options.

−i, −−in <FILE> [default: stdin]
The name of the FluxML (XML) input file. If omitted, the FluxML document is expected on standard input.

−m, −−mixture <FILE>
The file containing the substrate specification. See below for the format of this file.

−o, −−out <FILE> [default: stdout]
Name of an XML document (FluxML fragment) containing the optimized mixture. If this parameter is omitted the optimized substrate mixture is written to standard output.

−O, −−optimizer <PKG> [default: IPOPT]
The optimization algorithm to be used. The default is to use Ipopt. Other possible choices are CFSQP, NAGNLP, and SUBPLEX (CFSQP and NAGNLP are commercial and may not be accessible for your installation).

−P, −−properties <arg>
This option allows fine−grained control over the optimization algorithm's behavior and settings. The argument to option **−P** is a comma−separated list of key−value pairs. The general syntax is: **[optimizer].[property]=[type]([value])**.

Valid **[optimizer]**'s are '**ipopt**' and '**cfsqp**'.

Valid **[type]**'s are:

- **integer** − in this case **[value]** has to be an integer.
- **string** − the contents of **[value]** are allowed to be any string.
- **real** − for real (double precision) values.
- **boolean** − restricts the contents of **[value]** to the values **true** and **false**.

Using this interface all of the numerous settings of Ipopt can be accessed. A full list of valid options can be obtained by setting the boolean property **ipopt.print_options_documentstion** to **true**. See the examples below. Other interesting properties include:

- **ipopt.max_cpu_time** − allows to abort Ipopt after a specified number of CPU seconds is exceeded.
- **ipopt.print_level** − the verbosity of Ipopt. Valid settings are integers from 0 to 12. The default setting is 5.

- **ipopt.max_iter** − the maximum number of iterations before termination. The default value is 3000.
- **ipopt.linear_solver** − allows to choose the linear solver used by Ipopt. The default setting is '**mumps**'. Using another solver may improve convergence speed and optimization results.

CFSQP supports significantly fewer properties (a more detailled description can be found in the CFSQP documentation):

- **cfsqp.mode** − the type and mode of the used solver. The default setting is 200.
- **cfsqp.iprint** − the verbosity of CFSQP. The default setting is 1. Other settings are 0 (quiet), 2, or 3 (more verbose output).
- **cfsqp.miter** − the maximum number of iterations before termination. The default value is 1000.
- **cfsqp.bigbnd** − allows setting the value which plays the role of infinity. The default value is 1e20.
- **cfsqp.eps** − final norm of the newton gradient. Must be bigger than the machine epsilon. The default value is 1e−10.

NAGNLP refers to the "nag_opt_nlp" ("e04ucc") optimizer found in the commercial NAGC library. Most important settings are accessible via the following properties (cf. NAGC documentation):

- **nag_opt_nlp.max_iter** − the maximum number of iterations before termination. The default value is 250.
- **nag_opt_nlp.list** − print settings. The default value is "false".
- **nag_opt_nlp.print_level** − the major print level. The default setting is "Nag_Soln_Iter". See the NAGC documentation for other available settings.
- **nag_opt_nlp.minor_print_level** − the minor print level. The default setting is "Nag_NoPrint". See the NAGC documentation for other available settings.
- **nag_opt_nlp.verify_grad** − allows to enable the gradient checker. The default setting is "Nag_NoCheck". Set to "Nag_SimpleCheck" to turn on gradient checking.

−r, −−random <NUM> [default: 1]
The number of randomly initialized multi−start optimization runs. This option defaults to 1, i.e. single optimization run.

−C, −−criterion [ADEM|expr] [default : D]
This option allows to choose between different statistical optimality criteria / information indices computed from the covariance matrix of free fluxes. The four basic choices are (Atkinson and Donev: Optimum experimental designs. Oxford University Press, 1992):

- **A** (A−optimality): the value trace(C)/n, where trace(C) is the sum of the free fluxes' variances and n=dim(C) is the number of degrees of freedom (i.e. the number of free fluxes).
- **D** (D−optimality): the value det(C)ˆ(1/n), where det(C) is the determinant of the free fluxes' covariance matrix and n=dim(C) is the number of degrees of freedom (i.e. the number of free fluxes).
- **E** (E−optimality): the maximum eigenvalue of the covariance matrix (proportional to the length of the longest principal axis of the covariance ellipsoid).
- **M** (M−optimality): the maximum variance, i.e. the maximum diagonal element of the covariance matrix (the square of the largest confidence interval).

In addition to the four basic choices, this command line option allows to specify an arbitrary formula including also the following variables:

- **t**, the trace of the free fluxes' covariance matrix.
- **Dim**, the dimension of the full covariance matrix (including all fluxes).
- **dim**, the dimension of the covariance matrix. This value is identical to the number of the free fluxes and the degrees of freedom.
- **d**, the determinant of the free fluxes' covariance matrix.
- **e**, the minimum eigenvalue of the covariance matrix.
- **c**, the minimum confidence interval / standard deviation, i.e. the root of the minimum diagonal value.
- **S**, the maximum standard deviation, i.e. the root of the maximum diagonal value.
- **s**, the minimum standard deviation, i.e. the root of the minimum diagonal value.

Allowed operators include: +, −, *, /, ^, abs(x), sqr(x), sqrt(x,y), exp(x), log(x), log2(x), log10(x), min(x,y), max(x,y), =, !=, <, > <=, >=

−L, −−list
Specifying this option results in a list of allowed configuration names for the specified FluxML document. The program exits immediately after emitting the list.

−c, −−configure <CFG> [default: 'default']
Because FluxML documents may contain several **<configuration/>** elements this option allows to specify the configuration that should be used for the simulation. If this option is omitted it is assumed that the FluxML document contains a configuration with the name "default".

−l, −−log DEST
Specify the destination for the internal logging. In the most simple case **DEST** is a file name of a log file. In case the file exists new log messages are appended. Apart from log files it is possible to publish log messages to file descriptors, UNIX domain sockets, UDP and SCTP ports, and a small graphical user interface.

A file descriptor is specified by **fd:[num]**, where **[num]** is the number of the file descriptor.

A unix domain socket in the local file system is specified by **unix:[name]**, where **[name]** is the name of the socket file

A UDP or (connectionless) SCTP port is specified by **[proto]:[host]:[port]**, where **[proto]** is either "udp" or "sctp" and **[host]** is the name of the destination host and **[port]** is a UDP or SCTP port number on the destination host. Please note that the length of log messages is bounded by the minimum safe UDP packet size − log messages containing more than 548 characters will be truncated.

Finally, log messages can also be sent to a small GUI by specifying the destination **@gui@**. The GUI requires a working Perl/Tk installation and a running X server.

In order to capture all log messages concerning the command line processing this option should be specified in front of all other options.

−v, −−verbose 0..10 [default: 5]
Specify the verbosity 0, 1, ..., 10 of generated / emitted log messages. The meaning of the different log levels is as follows:

- **0 (QUIET)** do not emit log messages at all.
- **1 (ERROR)** only emit severe error messages.
- **2 (WARNING)** only report severe errors and warnings.

- **3 (NOTICE)** report all errors and warnings including important informal messages.
- **4 (INFO)** report all errors, warnings and all informal messages.
- **5 (THROW)** in case of an exception, try to give a diagnosis of the error; sometimes even gives a backtrace of the current function stack.
- **6 (DEBUG0)** emit the more important debugging messages.
- **7 (DEBUG1)** emit the less important debugging messages
- **8 (DEBUG2)** emit the superfluous debugging messages
- **9 (DEBUG3)** emit annoying debugging messages.
- **10 (DEBUG4)** don't dare to use it!

SOLVER OPTIONS

−a, −−apsolve
Solve the individual linear equation systems of the cumomer or EMU cascade using arbitrary precision arithmetic. Note that the results are converted to double precision immediately after solution. This option eliminates any round−off for the solution of the individual network levels.

−A, −−exact
In addition to option **−a** absolutely all computations are carried out using arbitrary precision arithmetic. If this option is used in conjunction with an analytical gradient (**−g analytic**) this results in exact derivatives. The results are converted to double precision just before writing them to the output file. This option is intended for debugging purposes. For large networks models the use of arbitrary precision arithmetic is probably to expensive.

−d, −−dbgsolve
When this option is used the network model is simulated in numerical debugging mode: for every equation system condition numbers are computed and the residual of the solution is checked.

MIXTURE SPECIFICATION

The XML document for mixture specification is part of the FluxML document format and collects an arbitrary number of <input/> elements for possible different substrate pools under one <mixture/> element. In order to allow a further description of the resulting mixture each of the <input/> is required to have an id attribute with unique value. Example (including corrections for natually occuring isotopes):

```
<?xml version="1.0"?>
<mixture xmlns="http://www.13cflux.net/fluxml">
  <input id="first_mix" pool="A" type="isotopomer">
    <label cfg="110" purity="0.98">1</label>
  </input>
  <input id="second_mix" pool="A" type="isotopomer">
    <label cfg="011" purity="0.96">1</label>
  </input>
  <input id="third_mix" pool="A" type="isotopomer">
    <label cfg="101" purity="0.99">1</label>
  </input>
</mixture>
```

EXAMPLES

Optimize a subtrate mixture 50 times starting from a randomized initialization:

```
edopt −r 50 −i network.fml −m mixture_in.mix −o mixture_out.mix
```

SEE ALSO

fitfluxes(1), edscanner(1)

AUTHOR

Michael Weitzel

This manpage was written by Michael Weitzel <mich@el−weitzel.de>.

NAME

edscanner − experimental design scanner (for statistical mixture quality).

SYNOPSIS

edscanner [*options*]

DESCRIPTION

Given a FluxML file and a specification of a mixture of different available labeling substrates the program **edscanner** samples the possibly high dimensional mixing simplex at evenly spaced points. At each sampling point a linearized statistical analysis is performed and the volume of the ellipsoid corresponding to the fluxes' covariance matrix is computed.

The coordinates of the sampling points including the volume information are written to a HDF5 file. A further analysis of this data may include the visualization of mixing triangles (using the provided MATLAB script drawMixingTriangle.m) or the description of the resulting statistical data using high−dimension density functions.

Be aware that successful experimental design may require to specify an error model the extrapolation of standard errors of simulated measurements. Details can be found in **fluxml**(5) (element "errormodel").

OPTIONS

−h, −−help
Show a brief help for all command line options.

−i, −−in <FILE> [default: stdin]
The name of the FluxML (XML) input file. If omitted, the FluxML document is expected on standard input.

−m, −−mixture <FILE>
The file containing the substrate specification. See below for the format of this file.

−o, −−out <FILE>
Name of an output HDF5 file. This option is required. The generated HDF5 file contains a single matrix with the path '/mix/matrix'. For n different mixing partners each row first contains the n−1 cartesian coordinates x_1, ..., x_{n−1}, followed by the n barycentric coordinates t_1, ..., t_n, and finally the information index (optimality criterion) of the covariance matrix (cf. option **−C**):

((x_1, ..., x_n−1, t_1, ..., t_n,)+ inf.index, cost)

Dataset '/mix/c' contains the column labels for matrix '/mix/matrix'. The value '/mix/central_value' contains the information index of the covariance matrix corresponding to the center of the mixing simplex, i.e. same proportions of all mixing partners.

−n, −−nsamples <NUM> [default: 10]
The number of samples to be generated. Defaults to 10.

−C, −−criterion [ADEM|expr] [default : D]
This option allows to choose between different statistical optimality criteria / information indices computed from the covariance matrix of free fluxes. The four basic choices are (Atkinson and Donev: Optimum experimental designs. Oxford University Press, 1992):

- **A** (A−optimality): the value trace(C)/n, where trace(C) is the sum of the free fluxes' variances and n=dim(C) is the number of degrees of freedom (i.e. the number of free fluxes).
- **D** (D−optimality): the value det(C)ˆ(1/n), where det(C) is the determinant of the free fluxes' covariance matrix and n=dim(C) is the number of degrees of freedom (i.e. the number of free fluxes).
- **E** (E−optimality): the maximum eigenvalue of the covariance matrix (proportional to the length of the longest principal axis of the covariance ellipsoid).
- **M** (M−optimality): the maximum variance, i.e. the maximum diagonal element of the covariance matrix (the square of the largest confidence interval).

In addition to the four basic choices, this command line option allows to specify an arbitrary formula including also the following variables:

- **t**, the trace of the free fluxes' covariance matrix.
- **Dim**, the dimension of the full covariance matrix (including all fluxes).
- **dim**, the dimension of the covariance matrix. This value is identical to the number of the free fluxes and the degrees of freedom.
- **d**, the determinant of the free fluxes' covariance matrix.
- **e**, the minimum eigenvalue of the covariance matrix.
- **c**, the minimum confidence interval / standard deviation, i.e. the root of the minimum diagonal value.
- **S**, the maximum standard deviation, i.e. the root of the maximum diagonal value.
- **s**, the minimum standard deviation, i.e. the root of the minimum diagonal value.

Allowed operators include: +, −, *, /, ^, abs(x), sqr(x), sqrt(x,y), exp(x), log(x), log2(x), log10(x), min(x,y), max(x,y), =, !=, <, > <=, >=

−L, −−list
Specifying this option results in a list of allowed configuration names for the specified FluxML document. The program exits immediately after emitting the list.

−c, −−configure <CFG> [default: 'default']
Because FluxML documents may contain several **<configuration/>** elements this option allows to specify the configuration that should be used for the simulation. If this option is omitted it is assumed that the FluxML document contains a configuration with the name "default".

−t, −−tolerance <val> [default: 1e−9]
Specifies a constraint violation tolerance value. This parameter can be used to tolerate a certain constraint violation. Use with care.

−l, −−log DEST
Specify the destination for the internal logging. In the most simple case **DEST** is a file name of a log file. In case the file exists new log messages are appended. Apart from log files it is possible to publish log messages to file descriptors, UNIX domain sockets, UDP and SCTP ports, and a small graphical user interface.

A file descriptor is specified by **fd:[num]**, where **[num]** is the number of the file descriptor.

A unix domain socket in the local file system is specified by **unix:[name]**, where **[name]** is the name of the socket file

A UDP or (connectionless) SCTP port is specified by **[proto]:[host]:[port]**, where **[proto]** is either "udp" or "sctp" and **[host]** is the name of the destination host and **[port]** is a UDP or SCTP port number on the destination host. Please note that the length of log messages is bounded by the minimum safe UDP packet size − log messages containing more than 548 characters will be truncated.

Finally, log messages can also be sent to a small GUI by specifying the destination **@gui@**. The GUI requires a working Perl/Tk installation and a running X server.

In order to capture all log messages concerning the command line processing this option should be specified in front of all other options.

−v, −−verbose 0..10 [default: 5]
Specify the verbosity 0, 1, ..., 10 of generated / emitted log messages. The meaning of the different log levels is as follows:

- **0 (QUIET)** do not emit log messages at all.
- **1 (ERROR)** only emit severe error messages.
- **2 (WARNING)** only report severe errors and warnings.
- **3 (NOTICE)** report all errors and warnings including important informal messages.
- **4 (INFO)** report all errors, warnings and all informal messages.
- **5 (THROW)** in case of an exception, try to give a diagnosis of the error; sometimes even gives a backtrace of the current function stack.
- **6 (DEBUG0)** emit the more important debugging messages.
- **7 (DEBUG1)** emit the less important debugging messages
- **8 (DEBUG2)** emit the superfluous debugging messages
- **9 (DEBUG3)** emit annoying debugging messages.
- **10 (DEBUG4)** don't dare to use it!

SOLVER OPTIONS

−a, −−apsolve

Solve the individual linear equation systems of the cumomer or EMU cascade using arbitrary precision arithmetic. Note that the results are converted to double precision immediately after solution. This option eliminates any round−off for the solution of the individual network levels.

−A, −−exact

In addition to option **−a** absolutely all computations are carried out using arbitrary precision arithmetic. If this option is used in conjunction with an analytical gradient (**−g analytic**) this results in exact derivatives. The results are converted to double precision just before writing them to the output file. This option is intended for debugging purposes. For large networks models the use of arbitrary precision arithmetic is probably to expensive.

−d, −−dbgsolve

When this option is used the network model is simulated in numerical debugging mode: for every equation system condition numbers are computed and the residual of the solution is checked.

MIXTURE SPECIFICATION

The XML document for mixture specification is part of the FluxML document format and collects an arbitrary number of <input/> elements for possible different substrate pools under one <mixture/> element. In order to allow a further description of the resulting mixture each of the <input/> is required to have an id attribute with unique value. Example (including corrections for natually occuring isotopes):

```
<?xml version="1.0"?>
<mixture xmlns="http://www.13cflux.net/fluxml">
  <input id="first_mix" pool="A" type="isotopomer">
    <label cfg="110" purity="0.98">1</label>
  </input>
  <input id="second_mix" pool="A" type="isotopomer">
    <label cfg="011" purity="0.96">1</label>
  </input>
  <input id="third_mix" pool="A" type="isotopomer">
    <label cfg="101" purity="0.99">1</label>
  </input>
</mixture>
```

EXAMPLES

Raster a possibly high dimensional mixing simplex with 500 evenly spaced samples. Carry−out all computations using arbitrary precision arithmetic:

edscanner −i network.fml −m mixture.mix −o samples.h5 −n 500 −A

In case there are only three mixing partners the result can be visualized in form of a triangle plot using the supplied MATLAB script drawMixingTriangle.m:

>> drawMixingTriangle('samples.h5');

See help drawMixingTriangle for more info.

SEE ALSO

edopt(1), fwdsim(1)

AUTHOR

Michael Weitzel

This manpage was written by Michael Weitzel <mich@el−weitzel.de>.

NAME

fitfluxes − fit a flux distribution of a network in order to reproduce a set of isotope labeling measurement values.

SYNOPSIS

fitfluxes [*options*]

DESCRIPTION

Fitfluxes is a program for intelligently adjusting a flux distribution of a metabolic network in order to reproduce a set of isotope labeling measurement values.

For this purpose **fitfluxes** reads the metabolic network and measurement configuration from a FluxML file, analyzes the network's stoichiometry and uses an optimization algorithm for adjusting certain 'free' variables in the flux distribution. Given the resulting new flux distribution the network and the measurements are simulated and the resulting synthetic measurement values are compared to the real ones. This process is repeated until synthetic and real measurement values agree or a certain termination condition is fulfilled (e.g. the maximum number of iterations is exceeded).

The resulting flux distribution and the corresponding synthetic measurement values are written to a **fwdsim**(5) XML document, i.e. a document in the same format also generated by the **fwdsim**(1) command.

COMMON OPTIONS

The following options are common to **fwdsim** and **fitfluxes**:

−h, −−help
Show a brief help for all command line options.

−i, −−in <FILE> [default: stdin]
The name of the FluxML (XML) input file. If omitted, the FluxML document is expected on standard input.

−o, −−out <FILE> [default: stdout]
The name of the FWDSIM (XML) output file. If omitted, the generated FWDSIM document is written to standard output.

−L, −−list
Specifying this option results in a list of allowed configuration names for the specified FluxML document. The program exits immediately after emitting the list.

−c, −−configure <CFG> [default: 'default']
Because FluxML documents may contain several **<configuration/>** elements this option allows to specify the configuration that should be used for the simulation. If this option is omitted it is assumed that the FluxML document contains a configuration with the name "default".

−l, −−log DEST
Specify the destination for the internal logging. In the most simple case **DEST** is a file name of a log file. In case the file exists new log messages are appended. Apart from log files it is possible to publish log messages to file descriptors, UNIX domain sockets, UDP and SCTP ports, and a small graphical user interface.

A file descriptor is specified by **fd:[num]**, where **[num]** is the number of the file descriptor.

A unix domain socket in the local file system is specified by **unix:[name]**, where **[name]** is the name of the socket file

A UDP or (connectionless) SCTP port is specified by **[proto]:[host]:[port]**, where **[proto]** is either "udp" or "sctp" and **[host]** is the name of the destination host and **[port]** is a UDP or SCTP port number on the destination host. Please note that the length of log messages is bounded by the minimum safe UDP packet size − log messages containing more than 548 characters will be truncated.

Finally, log messages can also be sent to a small GUI by specifying the destination **@gui@**. The GUI

requires a working Perl/Tk installation and a running X server.

In order to capture all log messages concerning the command line processing this option should be specified in front of all other options.

−v, −−verbose 0..10 [default: 5]
Specify the verbosity 0, 1, ..., 10 of generated / emitted log messages. The meaning of the different log levels is as follows:

- **0 (QUIET)** do not emit log messages at all.
- **1 (ERROR)** only emit severe error messages.
- **2 (WARNING)** only report severe errors and warnings.
- **3 (NOTICE)** report all errors and warnings including important informal messages.
- **4 (INFO)** report all errors, warnings and all informal messages.
- **5 (THROW)** in case of an exception, try to give a diagnosis of the error; sometimes even gives a backtrace of the current function stack.
- **6 (DEBUG0)** emit the more important debugging messages.
- **7 (DEBUG1)** emit the less important debugging messages
- **8 (DEBUG2)** emit the superfluous debugging messages
- **9 (DEBUG3)** emit annoying debugging messages.
- **10 (DEBUG4)** don't dare to use it!

−t, −−tolerance <val> [default: 1e−9]
Specifies a constraint violation tolerance value. This parameter can be used to tolerate a certain constraint violation. Use with care.

SOLVER OPTIONS

There are three options affecting the behavior of the solver. The same options are supported by the **fwdsim** command:

−a, −−apsolve
Solve the individual linear equation systems of the cumomer or EMU cascade using arbitrary precision arithmetic. Note that the results are converted to double precision immediately after solution. This option eliminates any round−off for the solution of the individual network levels.

−A, −−exact
In addition to option **−a** absolutely all computations are carried out using arbitrary precision arithmetic. If this option is used in conjunction with an analytical gradient (**−g analytic**) this results in exact derivatives. The results are converted to double precision just before writing them to the output file. This option is intended for debugging purposes. For large networks models the use of arbitrary precision arithmetic is probably to expensive.

−d, −−dbgsolve
When this option is used the network model is simulated in numerical debugging mode: for every equation system condition numbers are computed and the residual of the solution is checked.

OPTIMIZATION OPTIONS

−O, −−optimizer <arg> [default: IPOPT]
The desired optimization algorithm. Currently available are **Ipopt**, **CFSQP**, and **NAGNLP** (the latter two are distributed under a commercial license and may not be available at your site).

−P, −−properties <arg>
This option allows fine−grained control over the optimization algorithm's behavior and settings. The argument to option **−P** is a comma−separated list of key−value pairs. The general syntax is: **[optimizer].[property]=[type]([value])**.

Valid **[optimizer]**'s are '**ipopt**' and '**cfsqp**'.

Valid **[type]**'s are:

- **integer** − in this case **[value]** has to be an integer.
- **string** − the contents of **[value]** are allowed to be any string.
- **real** − for real (double precision) values.
- **boolean** − restricts the contents of **[value]** to the values **true** and **false**.

Using this interface all of the numerous settings of Ipopt can be accessed. A full list of valid options can be obtained by setting the boolean property **ipopt.print_options_documentstion** to **true**. See the examples below. Other interesting properties include:

- **ipopt.max_cpu_time** − allows to abort Ipopt after a specified number of CPU seconds is exceeded.
- **ipopt.print_level** − the verbosity of Ipopt. Valid settings are integers from 0 to 12. The default setting is 5.
- **ipopt.max_iter** − the maximum number of iterations before termination. The default value is 3000.
- **ipopt.linear_solver** − allows to choose the linear solver used by Ipopt. The default setting is '**mumps**'. Using another solver may improve convergence speed and optimization results.

CFSQP supports significantly fewer properties (a more detailled description can be found in the CFSQP documentation):

- **cfsqp.mode** − the type and mode of the used solver. The default setting is 200.
- **cfsqp.iprint** − the verbosity of CFSQP. The default setting is 1. Other settings are 0 (quiet), 2, or 3 (more verbose output).
- **cfsqp.miter** − the maximum number of iterations before termination. The default value is 1000.
- **cfsqp.bigbnd** − allows setting the value which plays the role of infinity. The default value is 1e20.
- **cfsqp.eps** − final norm of the newton gradient. Must be bigger than the machine epsilon. The default value is 1e−10.

NAGNLP refers to the "nag_opt_nlp" ("e04ucc") optimizer found in the commercial NAGC library. Most important settings are accessible via the following properties (cf. NAGC documentation):

- **nag_opt_nlp.max_iter** − the maximum number of iterations before termination. The default value is 250.
- **nag_opt_nlp.list** − print settings. The default value is "false".
- **nag_opt_nlp.print_level** − the major print level. The default setting is "Nag_Soln_Iter". See the NAGC documentation for other available settings.
- **nag_opt_nlp.minor_print_level** − the minor print level. The default setting is "Nag_NoPrint". See the NAGC documentation for other available settings.
- **nag_opt_nlp.verify_grad** − allows to enable the gradient checker. The default setting is "Nag_NoCheck". Set to "Nag_SimpleCheck" to turn on gradient checking.

−S, −−shrink <eps> [default: 5e−7]

This option may be used to tighten the complete set of constraints exposed to the optimization packages. If you think of the constraints as a convex polyhedron this option causes the polyhedron to be shrunk symmetrically about the specified argument eps. Specify this option if you notice warning

messages reporting that the optimizer is violating constraints. Because this seems to be a common problem in the used optimization packages (probably due to numerical inaccuracies) fitfluxes uses a default shrinking of 5−e7. Specify −S 0 to disable this feature.

−g, −−gradient <arg> [default: fd4]
The desired method for computaton of partial derivatives in case statistics are to be computed (option **−s**). Possible values are '**fd1**', '**fd2**', '**fd3**', '**fd4**' for finite difference approximations, and '**analytic**' for exact derivatives. The default value is '**fd4**', i.e. the gradient is approximated using an O(h^4) finite difference formula (which is slightly faster than using the exact derivatives).

EXAMPLES

Find a new flux distribution for a metabolic network specified in a FluxML file in order to reproduce a set of istope labeling measurement values. Safe the XML output to file network.fwd (most simple case):

fitfluxes −i network.fml −o network.fwd

Use the optimization package CFSQP instead of Ipopt and compute the required gradient analytically (instead of fourth order finite differences):

fitfluxes −i network.fml −o network.fwd −O CFSQP −g analytic

Convert an old FTBL to FluxML, find a new flux distribution using Ipopt, and filter out the flux distribution in net / echange01 coordinates (the flux coordinate system of 13CFLUX 1). Send all log messages of fitfluxes to a GUI (requires a running X server):

ftbl2fml −i network.ftbl | fitfluxes −l @gui@ | fwdsimflt −s −X

Request the documentation of all settings from Ipopt. Also set option **max_iter** to 0 in order to abort as soon as possible. Redirect the output to text file ipopt_settings.txt:

fitfluxes −i network.fml −o /dev/null −P "ipopt.print_options_documentation=boolean(true), ipopt.max_iter=integer(0)" > ipopt_settings.txt

Use the commercial NAG NLP SQP optimizer (nag_opt_nlp, e04ucc) and restrict the maximum number of iterations to 50 (default: 1000):

fitfluxes −i network.fml −o network.fwd −O nagnlp −P "nag_opt_nlp.max_iter=integer(50)"

Request a list of available options along with their default settings from the NAG NLP optimizer (the options and their meaning is discussed in the NAG manual of e04ucc):

fitfluxes −i network.fml −o /dev/null −O nagnlp −P "nag_opt_nlp.print_available_options=boolean(true)"

SEE ALSO

fwdsim(1), fwdsim(5), fwdsimflt(1), ftbl2fml(1), simreport(1), setfluxes(1), multifit(1)

AUTHOR

Michael Weitzel

This manpage was written by Michael Weitzel <mich@el−weitzel.de>.

NAME

fmllint − a lint tool (syntax checker) for FluxML.

SYNOPSIS

fmllint [*options*]

DESCRIPTION

The program **fmllint** is a simple FluxML validation tool. In addition to the syntactic check provided by the underlying Xerces−C XML Schema validation **fmllint** performs a thorough semantic check of a FluxML document. A name of a FluxML file or an URL may be passed on the command line. In case the command line is empty, the FluxML document is expected on standard input.

COMMON OPTIONS

−h, −−help
Show a brief help for all command line options.

−i, −−in <FILE> [default: stdin]
The name of the FluxML (XML) input file. If omitted, the FluxML document is expected on standard input.

−o, −−out <FILE> [default: no output]
The name of the output FluxML file. If this option is omitted no output is generated. Use "−" for generating output on standard output

−L, −−list
Specifying this option results in a list of allowed configuration names for the specified FluxML document. The program exits immediately after emitting the list.

−l, −−log DEST
Specify the destination for the internal logging. In the most simple case **DEST** is a file name of a log file. In case the file exists new log messages are appended. Apart from log files it is possible to publish log messages to file descriptors, UNIX domain sockets, UDP and SCTP ports, and a small graphical user interface.

A file descriptor is specified by **fd:[num]**, where **[num]** is the number of the file descriptor.

A unix domain socket in the local file system is specified by **unix:[name]**, where **[name]** is the name of the socket file

A UDP or (connectionless) SCTP port is specified by **[proto]:[host]:[port]**, where **[proto]** is either "udp" or "sctp" and **[host]** is the name of the destination host and **[port]** is a UDP or SCTP port number on the destination host. Please note that the length of log messages is bounded by the minimum safe UDP packet size − log messages containing more than 548 characters will be truncated.

Finally, log messages can also be sent to a small GUI by specifying the destination **@gui@**. The GUI requires a working Perl/Tk installation and a running X server.

In order to capture all log messages concerning the command line processing this option should be specified in front of all other options.

−v, −−verbose 0..10 [default: 5]
Specify the verbosity 0, 1, ..., 10 of generated / emitted log messages. The meaning of the different log levels is as follows:

- **0 (QUIET)** do not emit log messages at all.
- **1 (ERROR)** only emit severe error messages.
- **2 (WARNING)** only report severe errors and warnings.
- **3 (NOTICE)** report all errors and warnings including important informal messages.

- **4 (INFO)** report all errors, warnings and all informal messages.
- **5 (THROW)** in case of an exception, try to give a diagnosis of the error; sometimes even gives a backtrace of the current function stack.
- **6 (DEBUG0)** emit the more important debugging messages.
- **7 (DEBUG1)** emit the less important debugging messages
- **8 (DEBUG2)** emit the superfluous debugging messages
- **9 (DEBUG3)** emit annoying debugging messages.
- **10 (DEBUG4)** don't dare to use it!

SPECIAL OPTIONS

−C, −−challenge
Specifying this option results in the computation of a network−specific authentication challenge. This option and its result are reserved for internal use.

EXAMPLE

Check a local FluxML file for semantic and syntactic correctness:

fmllint −i network.fml

Check a remote file on a HTTP server for correctness:

fmllint −i http://www.13cflux.net/network.fml

SEE ALSO

xmllint(1)

AUTHOR

Michael Weitzel

This manpage was written by Michael Weitzel <mich@el−weitzel.de>.

NAME

ftbl2fml − create a FluxML file from an old FTBL file.

SYNOPSIS

ftbl2fml [*options*]

DESCRIPTION

Ftbl2fml converts a metabolic network model in FTBL file format (old 13CFLUX) into the new FluxML (XML) document format. This conversion includes the parametrization of the stoichiometry, constraint (in)equalities, measurement specifications and measurement data. Optionally, **ftbl2fml** will try to preserve the comments contained in the FTBL file.

Although **ftbl2fml** will, in general, do a good job the user of this program should be aware that there is a small semantic difference between FTBL and FluxML: 13CFLUX 2 and FluxML do not support 'output pools'. From the perspective of 13CFLUX 2 a metabolic network specification consists of

- substrate pools (aka 'input pools') carrying isotopic labeling information
- inner pools (aka 'intracellular pools'), from which a subset is required to be simulated in order to evaluate the measurement specifications.
- metabolic reactions (aka 'fluxes') connecting the inner pools.
- effluxes leaving the system and necessary to formulate the mass balance.

In addition, a network specification for the old 13CFLUX contains 'output pools' which collect all material transported by the effluxes. Because these output pools, on the one hand, violate mass conservation and, on the other hand, artificially increase the size of the network, output pools are no longer supported by 13CFLUX 2.

In **ftbl2fml**, output pools are automatically removed during conversion. Although this should be fine for most network specifications it results in an error if an isotopomer distribution of a removed output pool is required for the evaluation of a measurement specification. In case this problem occurs, it is necessary to reintroduce the output pool and an additional efflux, manually.

COMMON OPTIONS

−h, −−help
Show a brief help for all command line options.

−i, −−in <FILE> [default: stdin]
The name of the FTBL input file.

−o, −−out <FILE> [default: stdout]
The name of the FluxML (XML) output file.

−c, −−comments
Tries to preserve FTBL comments. FTBL comments are woven into the FluxML file in form of XML comments. As far as possible, this is done at the corresponding positions.

−d, −−unmapped
Preserves the comments of the unmapped sections of the FTBL file, i.e. the sections which are not converted into the FluxML file. This option requires option **−c**.

−e, −−effluxes
Preserves all output pools of the FTBL file by appending extra effluxes to them. The use of this option enlarges the network model, so that the simulation problem solved by 13CFLUX 2 is actually larger than the problem exposed to the old 13CFLUX. The default name of the newly introduced effluxes is 'efflux_' (see option **−e**) followed by the name of the corresponding output pool. Use this option with care.

−f, −−fprefix <PREFIX> [default: efflux_]
Specifies the prefix of the new effluxes introduced if option **−e** is given. The default prefix is 'efflux_'.

−F, −−freeconst
Transform the values of the free fluxes in the input FTBL file into constraints in the generated FluxML

document.

−k, −−noautoms
By default, and if applicable, the sums of labeling patterns in section LABEL_MEASUREMENTS are translated into compact MS specifications. The resulting MS specifications are easier to read and can be simulated with greater efficiency (EMU method). Although this translation should always be correct there might be an interest to retain the original sum formulas. In this case automatic translation can be turned of by specifying this parameter.

−l, −−listsinks
Create an XML comment containing the names of all removed output pools.

−m, −−mathml
Translate all constraint equalities and inequalities into the machine−readable Content−MathML. Since 13CFLUX 2 also allows a human−readable textual notation it should not be necessary to specify this parameter.

−n, −−nonpretty
Normally, **ftbl2fml** generates human−readable, pretty−printed FluxML. If no human intends to read / edit the resulting FluxML document this option may be specified.

−r, −−remsinks <REGEX>
Using this parameter it is possible to remove only selected output pools whose name matches a given regular expression. See regex(7) for the syntax of regular expressions. This option is intended for expert use only. Do not use it.

−t, −−tsfromfile
If this option is specified the timestamp in the FluxML info header is generated from the timestamp of the FTBL input file. If this option is omitted **ftbl2fml** tries to parse the timestamp in the FTBL file. This is often problematic since this timestamp does not follow a fixed syntax.

−v, −−valid
Create a FluxML document that can be validated. If this option is specified the generated FluxML document can be validated by external XML tools, such as xmllint(1). Since the FluxML parser will validate the generated FluxML document it is not explicitly necessary to specify this option.

−y, −−pypretty
This option enforces to use Python/minidom for pretty printing rather than the external tool xmllint(1), which is the default and results in prettier FluxML. In case xmllint(1) is not available Python/minidom is used automatically.

−z, −−gzip
Compress the generated FluxML document using the gzip(1) compression algorithm. Be aware that you have to specify an output file (option **−o**) whose name should end with '.gz' − **ftbl2fml** will not write compressed data to its stdout.

EXAMPLES

Convert an FTBL file into a FluxML file using the default settings (most simple case):

ftbl2fml −i network.ftbl −o network.fml

Convert an old FTBL to FluxML, simulate and filter out the simulated measurement values (**ftbl2fml** reads from stdin and writes to stdout):

ftbl2fml < network.ftbl | fwdsim | fwdsimflt −m

Convert an FTBL file into a compressed FluxML, preserve comments, list removed output pools, and encode all formulas using Content−MathML:

ftbl2fml −clmz −i network.ftbl −o network.fml.gz

SEE ALSO

xmllint(1), fwdsim(1), fmllint(1)

AUTHOR

Michael Weitzel

This manpage was written by Michael Weitzel <mich@el−weitzel.de>.

NAME

fwdsim − a tool for performing a forward simulation of an isotope labeling network.

SYNOPSIS

fwdsim [*options*]

DESCRIPTION

Fwdsim is a tool for simulating a FluxML specification of an isotope labeling network including all measurements.

For this purpose **fwdsim** reads the metabolic network and measurement configuration from a FluxML file, analyzes the network's stoichiometry and simulates the isotopic labeling distribution in order to provide synthetic measurement values. **fwdsim** allows to perform a (linearized) statistical analysis in order to assess the reliability and sensitivity of the current flux distribution.

The simulation results and the generated synthetic measurement values are written to a **fwdsim**(5) XML document. Apart from the XML output, **fwdsim** allows to export various data and symbolic equations to HDF5, MathML, and text documents.

COMMON OPTIONS

The following options are common to **fwdsim** and **fitfluxes**:

−h, −−help
Show a brief help for all command line options.

−i, −−in <FILE> [default: stdin]
The name of the FluxML (XML) input file. If omitted, the FluxML document is expected on standard input.

−o, −−out <FILE> [default: stdout]
The name of the FWDSIM (XML) output file. If omitted, the generated FWDSIM document is written to standard output.

−L, −−list
Specifying this option results in a list of allowed configuration names for the specified FluxML document. The program exits immediately after emitting the list.

−c, −−configure <CFG> [default: 'default']
Because FluxML documents may contain several **<configuration/>** elements this option allows to specify the configuration that should be used for the simulation. If this option is omitted it is assumed that the FluxML document contains a configuration with the name "default".

−l, −−log DEST
Specify the destination for the internal logging. In the most simple case **DEST** is a file name of a log file. In case the file exists new log messages are appended. Apart from log files it is possible to publish log messages to file descriptors, UNIX domain sockets, UDP and SCTP ports, and a small graphical user interface.

A file descriptor is specified by **fd:[num]**, where **[num]** is the number of the file descriptor.

A unix domain socket in the local file system is specified by **unix:[name]**, where **[name]** is the name of the socket file

A UDP or (connectionless) SCTP port is specified by **[proto]:[host]:[port]**, where **[proto]** is either "udp" or "sctp" and **[host]** is the name of the destination host and **[port]** is a UDP or SCTP port number on the destination host. Please note that the length of log messages is bounded by the minimum safe UDP packet size − log messages containing more than 548 characters will be truncated.

Finally, log messages can also be sent to a small GUI by specifying the destination **@gui@**. The GUI requires a working Perl/Tk installation and a running X server.

In order to capture all log messages concerning the command line processing this option should be specified in front of all other options.

−v, −−verbose 0..10 [default: 5]
Specify the verbosity 0, 1, ..., 10 of generated / emitted log messages. The meaning of the different log levels is as follows:

- **0 (QUIET)** do not emit log messages at all.
- **1 (ERROR)** only emit severe error messages.
- **2 (WARNING)** only report severe errors and warnings.
- **3 (NOTICE)** report all errors and warnings including important informal messages.
- **4 (INFO)** report all errors, warnings and all informal messages.
- **5 (THROW)** in case of an exception, try to give a diagnosis of the error; sometimes even gives a backtrace of the current function stack.
- **6 (DEBUG0)** emit the more important debugging messages.
- **7 (DEBUG1)** emit the less important debugging messages
- **8 (DEBUG2)** emit the superfluous debugging messages
- **9 (DEBUG3)** emit annoying debugging messages.
- **10 (DEBUG4)** don't dare to use it!

−t, −−tolerance <val> [default: 1e−9]
Specifies a constraint violation tolerance value. This parameter can be used to tolerate a certain constraint violation. Use with care.

SOLVER OPTIONS

There are three options affecting the behavior of the solver:

−a, −−apsolve
Solve the individual linear equation systems of the cumomer or EMU cascade using arbitrary precision arithmetic. Note that the results are converted to double precision immediately after solution. This option eliminates any round−off for the solution of the individual network levels.

−A, −−exact
In addition to option **−a** absolutely all computations are carried out using arbitrary precision arithmetic. If this option is used in conjunction with an analytical gradient (**−g analytic**) this results in exact derivatives. The results are converted to double precision just before writing them to the output file. This option is intended for debugging purposes. For large networks models the use of arbitrary precision arithmetic is probably to expensive.

−d, −−dbgsolve
When this option is used the network model is simulated in numerical debugging mode: for every equation system condition numbers are computed and the residual of the solution is checked.

−n, −−dry−run
Performs a dry−run, i.e. aborts the application after preprocessing of the input file and stoichiometry.

STATISTICS OPTIONS

fwdsim allows to evaluation linearized statistics.

−s, −−statistics
Compute linearized statistics for the current flux setting. This option is most beneficial if the flux setting is already optimized, i.e. results from an invocation of **fitfluxes**. Indeterminable fluxes will be reported. For all determinable fluxes standard deviations will be written to the output file (**−o**). If specified together with option **−H** (HDF5 export) the Jacobian of the measurements as well as the covariance matrix of the fluxes are exported into the HDF5 file.

−g, −−gradient <arg> [default: fd4]

The desired method for computaton of partial derivatives in case statistics are to be computed (option **−s**). Possible values are '**fd1**', '**fd2**', '**fd3**', '**fd4**' for finite difference approximations, and '**analytic**' for exact derivatives. The default value is '**fd4**', i.e. the gradient is approximated using an O(hˆ4) finite difference formula (which is slightly faster than using the exact derivatives).

DATA EXPORT OPTIONS

fwdsim allows to export the preprocessed stoichiometry, the generated system matrices, the symbolic system equations, the Jacobian of measurements, and covariance matrices into HDF5 and text files. A fully−fledged simulator−code for Octave/MATLAB can be exported into a .m file.

−H, −−hdf5 <FILE>

Export the stoichiometry and the left and right hand sides of the Cumomer / EMU equation systems into a HDF5 file. If, in addition, the computation of linearized statistics is requested (by option **−s**) the Jacobian of the measurements and the covariance matrix of the fluxes are exported, as well.

The exported data in the HDF5 files is intended to be used in MATLAB, but may also be used in other scientific software, such as Octave. The data in HDF5 files is organized in a directory−like structure. For example, in order to load the stoichiometric matrix out of the generated HDF5 file in MATLAB, the following command may be used:

```
S = hdf5read('file.h5','/stoichiometry/matrix');
```

Currently the following data is exported:

- '/stoichiometry/matrix': The stoichiometric matrix
- '/stoichiometry/r': The row labels of the stoichiometric matrix, i.e. string values. Assuming the row labels are imported into a MATLAB array 'rows' the proper way to access the k'th label is 'rows(k).Data'.
- '/stoichiometry/c': The column labels of the stoichiometric matrix.
- '/stoichiometry/fluxes': The flux distribution in net / exchange coordinates including the flux types. Possible flux types are:
  - FREE (free): the flux value may be chosen freely. In **fitfluxes**, these fluxes are adjusted by the optimization algorithm.
  - CONS (constraint): the flux value has a constant value. In **fitfluxes**, these fluxes must not be adjusted by the optimization algorithm.
  - DEPD (dependent): the flux value is determined by a number of constraint and free fluxes.
  - QCON (quasi−constraint): the flux value is completely determined by constraint fluxes.

  The first two columns in '/stoichiometry/fluxes' contain the values of the net and exchange fluxes. The remaining columns are boolean flags indicating the type of the type of the net and exchange flux.
- '/stoichiometry/r_fluxes': The row labels of the matrix '/stoichiometry/fluxes', i.e. the flux names.
- '/stoichiometry/c_fluxes': The column labels of the matrix '/stoichiometry/fluxes'. If the column label has the suffix '.n', it refers the net flux (e.g. FREE.n). The suffix '.x' flags the exchange flux (e.g. QCON.x).
- '/stoichiometry/kernel/matrix_net' and '/stoichiometry/kernel/matrix_xch': The kernel matrices for net and exchange fluxes. Given the values of the free net and exchange fluxes the kernel matrices are used for recomputing the values of the dependent fluxes. For a kernel matrix K, this is achieved by evaluating v = K*[1; v_f];, where v_f contains the values of the free fluxes.

Because 13CFLUX 2 determines kernel matrices using arbitrary precision arithmetic, the kernel matrices are the exact solutions of the stoichiometric system.

- '/stoichiometry/kernel/r_net' and '/stoichiometry/kernel/r_xch': The row labels of the kernel matrices for net and exchange fluxes, i.e. all flux values.
- '/stoichiometry/kernel/c_net' and '/stoichiometry/kernel/c_xch': The column labels of the kernel matrices for net and exchange fluxes, i.e. the names of the free net and free exchange fluxes. The label of the first column always contains the label '1', flagging that the first column of the kernel matrices contain the particular solution.

In case option **−s** is passed additional statistical data is written to the HDF5 files:

- '/cov_free/matrix': The covariance matrix of the free fluxes.
- '/cov_free/r' and '/cov_free/c': The row and column labels of the covariance matrix, i.e. the names of the free fluxes. Net and exchange fluxes are distinguished by the suffix '.n' or '.x'.
- '/cov/matrix': The full covariance matrix of all fluxes.
- '/cov/r' and '/cov/c': The row and column labels of the full covariance matrix.
- '/jacobian/matrix': The Jacobian of the measurements, i.e. partial derivatives of the measurement values with respect to the free fluxes.
- '/jacobian/r': The row labels of the Jacobian, i.e. names of the measurement values. The names of the measurement values are given in short notation (see bibliography).
- '/jacobian/c': The column labels of the Jacobian, i.e. the names of the free fluxes. Net and exchange fluxes are distinguished by the suffix '.n' or '.x'.

−M, −−mathml <FILE>
Export the cascade's symbolic systems into a MathML file. The resulting Content−MathML file may be imported in computer algebra systems like Maple and Mathematica.

−T, −−text <FILE>
Exports the cumomer / EMU cascade's symbolic equation system into the specified text file.

−m, −−matlab <FILE>
Generate an Octave/MATLAB simulator function for the current network in the specified text file. The function name will match the name of the text file. The external function cumulate is contained in the 13CFLUX 2 installation.

EXAMPLES

Simulate an isotope labeling network and safe the XML output to file network.fwd (most simple case):

fwdsim −i network.fml −o network.fwd

Convert an old FTBL to FluxML, simulate and filter out the simulated measurement values (**fwdsim** reads from stdin and writes to stdout):

ftbl2fml −i network.ftbl | fwdsim | fwdsimflt −m

Simulate an isotope labeling network and generate all possible output; send the XML output to /dev/null:

fwdsim −i net.fml −o /dev/null −M m.mml −T t.txt −m f.m −H net.h5

Simulate an isotope labeling network and and compute linearized statistics. In addition to the simulation results, write estimated standard deviations to the output file. Save the stoichiometry, the cascaded equation systems, the Jacobian of measurements, and the covariance matrices to a HDF5 file:

fwdsim −s −i network.fml −o network.fwd −H network.h5

SEE ALSO

fitfluxes(1), fwdsimflt(1), ftbl2fml(1), simreport(1)

AUTHOR

Michael Weitzel

This manpage was written by Michael Weitzel <mich@el−weitzel.de>.

NAME

fwdsim2csv − transfer of flux settings from multiple FWDSIM (XML) documents into one CSV file

SYNOPSIS

fwdsim2csv [*options*] [*FWDSIM file names*]

DESCRIPTION

The script **fwdsim2csv** is used to transfer the flux settings from one or multiple FWDSIM (XML) document, possibly generated by the commands **fitfluxes**(1) and **multifit**(1) into human−readable CSV data. The CSV data is always written to standard output.

COMMON OPTIONS

−h, −−help
Show a brief help for all command line options.

OUTPUT OPTIONS

−s, −−separator <str> [default: ","]
The CSV field separator to use.

−a, −−all
Include all fluxes in the output, not just the free fluxes.

−n, −−net−only
Output only net fluxes and omit the exchange fluxes.

−x, −−xch−only
Output only exchange fluxes and omit the net fluxes.

−H, −−no−headers
Omit all column headers.

−R, −−no−residual
Omit the column containing the obtained residuals.

−p, −−opt−flags
Include additional columns containing the optimization flags.

−f, −−filenames
Include a column containing the file names of the FWDSIM files.

EXAMPLES

Read multiple FWDSIM (XML) files and output the values of all (free and dependent) net fluxes, and include the file names:

fwdsim2csv −anf *.fwd > fitdata.csv

Read one FWDSIM (XML) file from standard input and output the values of the free net and exchange fluxes, excluding the residuals:

fwdsim2csv −R < fit.fwd > fitdata.csv

SEE ALSO

fwdsim(1), fitfluxes(1), multifit(1)

AUTHOR

Michael Weitzel

This manpage was written by Michael Weitzel <mich@el−weitzel.de>.

NAME

fwdsimflt − filter the output of the FWDSIM (XML) documents generated by **fwdsim**(1) and **fitfluxes**(1)

SYNOPSIS

fwdsimflt [*options*]

DESCRIPTION

The tool **fwdsimflt** (fwdsim filter) can be used to filter the FWDSIM (XML) documents generated by the commands **fwdsim**(1) and **fitfluxes**(1) to a human−readable CSV format. In general, all information filtered by **fwdsimflt** is sent to stdout.

COMMON OPTIONS

−h, −−help
Show a brief help for all command line options.

−i, −−input <FILE> [default: stdin]
The name of the input FWDSIM (XML) file. If omitted, the FWDSIM document is expected on standard input.

FILTER OPTIONS

−m, −−measurements
Filter out simulated measurement values. The first column contains the index of the measurement value. The second column contains the simulated measurement value itself. The rows with the special index 0 contain a heading:

- column two contains the ID of the measurement group.
- column three contains the norm for the measurement group.
- the fourth column contains the short notation of the measurement specification.

−s, −−scompact
Specifying this option generates a list of flux values. The first column contains the flux name, the second and third column contain the value of the net and exchange flux.

−S, −−sfull
Output the full information about the stoichiometry. In particular, this includes:

1. the flux name.
2. the type of the net flux ('f'=free, 'd'=dependent, 'c'=constraint, 'q'=quasi−constraint).
3. the value of the net flux.
4. the symbolic equation of the net flux.
5. the type of the exchange flux.
6. the value of the exchange flux.
7. the symbolic equation of the net flux.

−t, −−stddevs
Output the standard deviations (if this information is contained in the input document).

−u, −−unknowns
Output all computed unknowns in either Cumomer or EMU coordinates. The output depends on the simulation method and type of network reduction specified in the original FluxML document.

−O, −−optimflags
Output used optimization flags and sensitivities.

FILTER-FILTER OPTIONS

The output generated for the above options can be further filtered by a number of filter−filter options:

−f, −−filter <REGEX>
Only output the data sets which flux name, pool name, or measurement group name is matched by the specified regular expression. See **regex**(7) for the syntax of regular expressions.

−F, −−fwdbwd
Transform the flux values (**−s, −S**) into forward / backward coordinates.

−X, −−netxch01
Transform the flux values (**−s, −S**) into net / exchange−[0,1] coordinates. This flux coordinate system is used by the old 13CFLUX software.

−U, −−isotopomers
Transform the unknowns from Cumomer or EMU coordinates (**−u**) into isotopomer fractions. Note that this is not always possible if a reduced network is simulated, i.e. not all Cumomers or EMUs were computed. If you need a full listing of all isotopomer fractions modify the underlying FluxML document in order to perform a 'full' simulation.

EXAMPLES

Read from file network.fwd and output all computed measurement values (most simple case):

fwdsimflt −i network.fwd −m

Read from stdin, filter output for measurement groups A, B, C, and G:

fwdsim −i network.fml | fwdsimflt −m −f "[A−C]" −f G

Read from file, output simulated unknowns of pools A and F:

fwdsimflt −u −f A −f F −i network.fwd

Read from file, output full stoichiometry:

fwdsimflt −S −i network.fwd

Read from stdin, output flux names, net− and exchange−[0,1] values:

fwdsimflt −s −X < network.fwd

SEE ALSO

fwdsim(1), fitfluxes(1)

AUTHOR

Michael Weitzel

This manpage was written by Michael Weitzel <mich@el−weitzel.de>.

NAME

hdf5tocsv − convert an XML file to a CSV formatted output.

SYNOPSIS

hdf5tocsv [*options*]

DESCRIPTION

Hdf5tocsv converts double matrices or vectors in HDF5 files from 13cflux(5) programs such as **ssampler(1)**, **collectfitdata(1)**, **multifwdsim(1)**, **multifitfluxes(1)**, or **multiperturb(1)** into a human−readable comma−separated text format (CSV). For instance, this is useful in situations where third−party tools have no option for processing HDF5 files.

COMMON OPTIONS

−h, −−help
Show a brief help for all command line options.

−i, −−in <FILE>
Path to HDF5 input file containing matrix data (−−data) and column texts(−−columns).

−o, −−out <FILE> [default: stdout]
Output CSV file. Unless specified with −−no−header, the first line contains the header column texts.

−D, −−data <H5FIELD> [default: /flux/data]
HDF5 path to the data matrix in the HDF5 file.

−C, −−columns <H5FIELD> [default: /flux/names]
HDF5 path to the header column texts in the HDF5 file.

SPECIAL OPTIONS

−d, −−csv−delimiter <CHAR> [default: ',']
Select a different separator character in the output CSV file.

−H, −−no−header
If set, the output of the column texts is omitted. This is useful for concatenating multiple HDF5 files into a single CSV output.

EXAMPLES

[source,shell]:

hdf5tocsv −i samples.hdf5 −o samples.csv

SEE ALSO

ssampler(1), collectfitdata(1), multifwdsim(1), multifwdsim(1), multiperturb(1), h5dump(1)

AUTHOR

This manpage was written by Tolga Dalman <info@13cflux.net> and Michael Weitzel <mich@el−weitzel.de>.

NAME

mcbootstrap − nonlinear statistical assessment of flux maps by Monte Carlo bootstrap

SYNOPSIS

mcbootstrap [*options*]

DESCRIPTION

mcbootstrap is a parallel implementation of the Monte Carlo bootstrap algorithm employing the 13CFLUX2 tools **ssampler(1)**, **perturb(1)**, **multifit(1)** and **collectfitdata(1)** as described in Dalman et al. FGCS, 2011, 10.1016/j.future.2011.10.007.

COMMON OPTIONS

−h, −−help
Show a brief help for all command line options.

−i, −−in <FILE> [default: stdin]
The name of the FluxML (XML) input file. If omitted, the FluxML document is expected on standard input.

−o, −−out <PATH> [default: stdout]
[default: .] Output directory for FWDSIM (XML) files. If omitted, the simulated FWDSIM files are placed in the current directory.

−c, −−configure <CFG> [default: 'default']
Because FluxML documents may contain several **<configuration/>** elements this option allows to specify the configuration that should be used for the simulation. If this option is omitted it is assumed that the FluxML document contains a configuration with the name "default".

−n, −−samples <NUM> [default: 1]
Number of random initial flux vectors generated with **ssampler(1)**

−v, −−verbose 0..10 [default: 5]
Specify the verbosity 0, 1, ..., 10 of generated / emitted log messages. The meaning of the different log levels is as follows:

- **0 (QUIET)** do not emit log messages at all.
- **1 (ERROR)** only emit severe error messages.
- **2 (WARNING)** only report severe errors and warnings.
- **3 (NOTICE)** report all errors and warnings including important informal messages.
- **4 (INFO)** report all errors, warnings and all informal messages.
- **5 (THROW)** in case of an exception, try to give a diagnosis of the error; sometimes even gives a backtrace of the current function stack.
- **6 (DEBUG0)** emit the more important debugging messages.
- **7 (DEBUG1)** emit the less important debugging messages
- **8 (DEBUG2)** emit the superfluous debugging messages
- **9 (DEBUG3)** emit annoying debugging messages.
- **10 (DEBUG4)** don't dare to use it!

EXAMPLES

[source,shell]:

mcbootstrap −i model.fml −o ouput_folder −n 2 −m 3

SEE ALSO

fluxml(5), fwdsim(5), fwdsim(1),ssampler(1),perturb(1), multifit(1), collectfitdata(1)

AUTHOR

This manpage was written by Tolga Dalman <info@13cflux.net> and Michael Weitzel <mich@el−weitzel.de>.

NAME

multifit − a multi−start, multi−processor frontend for fitfluxes.

SYNOPSIS

multifit [*options*] [−− *[additional parameters for fitfluxes]*]

DESCRIPTION

The program **multifit** runs a multi−processor, multi−start optimization using **fitfluxes**(1) based on random flux distributions created by the **ssampler**(1) tool. **multifit** performs a simple type of load balancing in order to use the available computational resource as efficient as possible.

Via command line option **−g, −−generate** it is possible to generate a configuration file which can be used to customize the command lines for preprocessing (sampling), processing (parameter fitting), and postprocessing (report generation). As soon as the preprocessing is finished it is safe to interrupt **multifit** by pressing Ctrl−C. In this case **multifit** will wait for the pending jobs to finish. After all jobs have finished, the index of the last finished job will be reported. This number may be used to restart **multifit** later (option **−r**).

All extra options specified after −− are directly passed to the **fitfluxes** invocations.

OPTIONS

−h, −−help
Show a brief help for all command line options.

−i, −−in <FILE> [default: stdin]
The name of the FluxML (XML) input file. If omitted, the FluxML document is expected on standard input.

−o, −−outdir <DIR> [default: ./]
Name of output directory. If none is specified the current directory is used. If the specified output directory does not exist it is created first.

−p, −−prefix <PKG> [default: IPOPT]
A prefix for the output file names written to the output directory. If no prefix is given, the prefix defaults to the name of the FluxML file.

−c, −−configure <CFG> [default: 'default']
Because FluxML documents may contain several **<configuration/>** elements this option allows to specify the configuration that should be used for the simulation. If this option is omitted it is assumed that the FluxML document contains a configuration with the name "default".

−n, −−samples <NUMBER>
The number of random flux distribution to generate. This equals to the number of fitfluxes jobs. This option is required if option **−f** is omitted or the file specified by option **−f** does not exist

−f, −−fluxdist <FILE>
The name of a HDF5 file containing random flux distributions. If the specified file does not exist it will be created. If this option is omitted a temporary file is created and erased on exit.

−t, −−tasks <RANGE> [default: <automatically determined>]
This option specifies a range for the number of parallel paths. If this option is not specified the number of parallel tasks is set to the number of currently idle processors. Possible values for <RANGE> are:

- **N** − where N is a fixed number of parallel tasks. This number is not changed during the processing.
- **N:** − where N is a lower bound for the number of parallel tasks. This number may be increased depending on the number of installed processors and current load of the machine.
- **:N** − where N is an upper bound of the number of parallel tasks. This number may be decreased depending on the number of installed processors and current load of the machine.

- **M:N** − where M and N are lower and upper bounds for the number of parallel tasks. Within these bounds, the number of parallel tasks may be adjusted depeneding on the number of installed processors and current load of the machine.

−r, −−restart <INDEX>
This option causes **multifit** to restart at a given dataset/job index and is useful if **multifit** was previously interrupted by pressing Ctrl−C.

−v, −−verbose
Echo task−id and command line of any executed command. This option is intended for debugging purposes.

−g, −−generate
When passing this option a template configuration file '$HOME/.x3cflux/.multifitrc' is generated. The configuration file may be used to adjust the command lines for sampling and parameter fitting.

−m, −−manpage
Displays an old, no longer maintained version of this manual page.

EXAMPLES

Run 100 multi−start optimizations on parallal on all free CPU cores. Save results to directory 'foobar' into files having prefix 'network'. Pass options '−O IPOPT −P ipopt.print_level=integer\(0\)' directly to **fitfluxes**(1):

multifit −i network.fml −n 100 −o foobar −p network −− −O IPOPT −P ipopt.print_level=integer\(0\)

SEE ALSO

ssampler(1), fitfluxes(1), setfluxes(1), collectfitdata(1)

AUTHOR

Michael Weitzel

This manpage was written by Michael Weitzel <mich@el−weitzel.de>.

NAME

multifitfluxes − parallel 13CFLUX2 parameter estimation tool

SYNOPSIS

multifitfluxes [*options*]

DESCRIPTION

Multifitfluxes is a parallel implementation of fitfluxes(1). Like the original **fitfluxes(1)** application, FluxML documents are taken as input along with several parameters. Unlike **fitfluxes(1)** , the output is either a directory eventually containing the result files (i.e., FWDSIM files containing parameter estimation results), or an unified HDF5 comprising flux values.

COMMON OPTIONS

−h, −−help
Show a brief help for all command line options.

−i, −−in <FILE> [default: stdin]
The name of the FluxML (XML) input file. If omitted, the FluxML document is expected on standard input.

−o, −−out <PATH> [default: stdout]
Output directory for FWDSIM (XML) files.

−c, −−configure <CFG> [default: 'default']
Because FluxML documents may contain several **<configuration/>** elements this option allows to specify the configuration that should be used for the simulation. If this option is omitted it is assumed that the FluxML document contains a configuration with the name "default".

−H, −−hdf5−in <File>
Input file containing flux samples (HDF5)

−f, −−hdf5−out <File>
Output file containing flux samples (HDF5)

−N, −−max−procs <NUM> [default: 1]
Number of maximum parallel processes

−B, −−block−size <NUM> [default: auto]
Number of samples per task. This option controls the parallelism granularity

−l, −−log DEST
Specify the destination for the internal logging. In the most simple case **DEST** is a file name of a log file. In case the file exists new log messages are appended. Apart from log files it is possible to publish log messages to file descriptors, UNIX domain sockets, UDP and SCTP ports, and a small graphical user interface.

A file descriptor is specified by **fd:[num]**, where **[num]** is the number of the file descriptor.

A unix domain socket in the local file system is specified by **unix:[name]**, where **[name]** is the name of the socket file

A UDP or (connectionless) SCTP port is specified by **[proto]:[host]:[port]**, where **[proto]** is either "udp" or "sctp" and **[host]** is the name of the destination host and **[port]** is a UDP or SCTP port number on the destination host. Please note that the length of log messages is bounded by the minimum safe UDP packet size − log messages containing more than 548 characters will be truncated.

Finally, log messages can also be sent to a small GUI by specifying the destination **@gui@**. The GUI requires a working Perl/Tk installation and a running X server.

In order to capture all log messages concerning the command line processing this option should be specified in front of all other options.

−v, −−verbose 0..10 [default: 5]
Specify the verbosity 0, 1, ..., 10 of generated / emitted log messages. The meaning of the different log levels is as follows:

- **0 (QUIET)** do not emit log messages at all.
- **1 (ERROR)** only emit severe error messages.
- **2 (WARNING)** only report severe errors and warnings.
- **3 (NOTICE)** report all errors and warnings including important informal messages.
- **4 (INFO)** report all errors, warnings and all informal messages.
- **5 (THROW)** in case of an exception, try to give a diagnosis of the error; sometimes even gives a backtrace of the current function stack.
- **6 (DEBUG0)** emit the more important debugging messages.
- **7 (DEBUG1)** emit the less important debugging messages
- **8 (DEBUG2)** emit the superfluous debugging messages
- **9 (DEBUG3)** emit annoying debugging messages.
- **10 (DEBUG4)** don't dare to use it!

−t, −−tolerance <val> [default: 1e−9]
Specifies a constraint violation tolerance value. This parameter can be used to tolerate a certain constraint violation. Use with care.

SOLVER OPTIONS

−a, −−apsolve
Solve the individual linear equation systems of the cumomer or EMU cascade using arbitrary precision arithmetic. Note that the results are converted to double precision immediately after solution. This option eliminates any round−off for the solution of the individual network levels.

−A, −−exact
In addition to option **−a** absolutely all computations are carried out using arbitrary precision arithmetic. If this option is used in conjunction with an analytical gradient (**−g analytic**) this results in exact derivatives. The results are converted to double precision just before writing them to the output file. This option is intended for debugging purposes. For large networks models the use of arbitrary precision arithmetic is probably to expensive.

−d, −−dbgsolve
When this option is used the network model is simulated in numerical debugging mode: for every equation system condition numbers are computed and the residual of the solution is checked.

OPTIMIZATION OPTIONS

−O, −−optimizer <arg> [default: IPOPT]
The desired optimization algorithm. Currently available are **Ipopt**, **CFSQP**, and **NAGNLP** (the latter two are distributed under a commercial license and may not be available at your site).

−P, −−properties <arg>
This option allows fine−grained control over the optimization algorithm's behavior and settings. The argument to option **−P** is a comma−separated list of key−value pairs. The general syntax is: **[optimizer].[property]=[type]([value])**.

Valid **[optimizer]**'s are '**ipopt**' and '**cfsqp**'.

Valid **[type]**'s are:

- **integer** − in this case **[value]** has to be an integer.
- **string** − the contents of **[value]** are allowed to be any string.

- **real** − for real (double precision) values.
- **boolean** − restricts the contents of **[value]** to the values **true** and **false**.

Using this interface all of the numerous settings of Ipopt can be accessed. A full list of valid options can be obtained by setting the boolean property **ipopt.print_options_documentstion** to **true**. See the examples below. Other interesting properties include:

- **ipopt.max_cpu_time** − allows to abort Ipopt after a specified number of CPU seconds is exceeded.
- **ipopt.print_level** − the verbosity of Ipopt. Valid settings are integers from 0 to 12. The default setting is 5.
- **ipopt.max_iter** − the maximum number of iterations before termination. The default value is 3000.
- **ipopt.linear_solver** − allows to choose the linear solver used by Ipopt. The default setting is '**mumps**'. Using another solver may improve convergence speed and optimization results.

CFSQP supports significantly fewer properties (a more detailled description can be found in the CFSQP documentation):

- **cfsqp.mode** − the type and mode of the used solver. The default setting is 200.
- **cfsqp.iprint** − the verbosity of CFSQP. The default setting is 1. Other settings are 0 (quiet), 2, or 3 (more verbose output).
- **cfsqp.miter** − the maximum number of iterations before termination. The default value is 1000.
- **cfsqp.bigbnd** − allows setting the value which plays the role of infinity. The default value is 1e20.
- **cfsqp.eps** − final norm of the newton gradient. Must be bigger than the machine epsilon. The default value is 1e−10.

NAGNLP refers to the "nag_opt_nlp" ("e04ucc") optimizer found in the commercial NAGC library. Most important settings are accessible via the following properties (cf. NAGC documentation):

- **nag_opt_nlp.max_iter** − the maximum number of iterations before termination. The default value is 250.
- **nag_opt_nlp.list** − print settings. The default value is "false".
- **nag_opt_nlp.print_level** − the major print level. The default setting is "Nag_Soln_Iter". See the NAGC documentation for other available settings.
- **nag_opt_nlp.minor_print_level** − the minor print level. The default setting is "Nag_NoPrint". See the NAGC documentation for other available settings.
- **nag_opt_nlp.verify_grad** − allows to enable the gradient checker. The default setting is "Nag_NoCheck". Set to "Nag_SimpleCheck" to turn on gradient checking.

−S, −−shrink <eps> [default: 5e−7]
This option may be used to tighten the complete set of constraints exposed to the optimization packages. If you think of the constraints as a convex polyhedron this option causes the polyhedron to be shrunk symmetrically about the specified argument eps. Specify this option if you notice warning messages reporting that the optimizer is violating constraints. Because this seems to be a common problem in the used optimization packages (probably due to numerical inaccuracies) fitfluxes uses a default shrinking of 5−e7. Specify −S 0 to disable this feature.

−g, −−gradient <arg> [default: fd4]
The desired method for computaton of partial derivatives in case statistics are to be computed (option **−s**). Possible values are '**fd1**', '**fd2**', '**fd3**', '**fd4**' for finite difference approximations, and '**analytic**' for

exact derivatives. The default value is '**fd4**', i.e. the gradient is approximated using an O(hˆ4) finite difference formula (which is slightly faster than using the exact derivatives).

EXAMPLES

[source,shell]:

multifitfluxes −i model.fml −H samples.hdf5 −f output.hdf5

SEE ALSO

fluxml(5), fitfluxes(5), fwdsim(1),

AUTHOR

Multifitfluxes extends fitfluxes(1), which was originally written by Michael Weitzel.

This manpage was written by Tolga Dalman <info@13cflux.net> and Michael Weitzel <mich@el−weitzel.de>.

NAME

multifwdsim − parallel 13CFLUX2 forward simulation tool

SYNOPSIS

multifwdsim [*options*]

DESCRIPTION

Multifwdsim is a parallel implementation of **fwdsim(1)** . Like the original **fwdsim(1)** application, FluxML documents are taken as input along with several parameters. Unlike **fwdsim(1)**, the output is a directory containing the result files (i.e., FWDSIM files containing simulation results)

COMMON OPTIONS

−h, −−help
Show a brief help for all command line options.

−i, −−in <FILE> [default: stdin]
The name of the FluxML (XML) input file. If omitted, the FluxML document is expected on standard input.

−o, −−out <PATH> [default: stdout]
Output directory for FWDSIM (XML) files.

−c, −−configure <CFG> [default: 'default']
Because FluxML documents may contain several **<configuration/>** elements this option allows to specify the configuration that should be used for the simulation. If this option is omitted it is assumed that the FluxML document contains a configuration with the name "default".

−H, −−hdf5−in <File>
Input file containing flux samples (HDF5)

−N, −−max−procs <NUM> [default: 1]
Number of maximum parallel processes

−B, −−block−size <NUM> [default: auto]
Number of samples per task. This option controls the parallelism granularity

−l, −−log DEST
Specify the destination for the internal logging. In the most simple case **DEST** is a file name of a log file. In case the file exists new log messages are appended. Apart from log files it is possible to publish log messages to file descriptors, UNIX domain sockets, UDP and SCTP ports, and a small graphical user interface.

A file descriptor is specified by **fd:[num]**, where **[num]** is the number of the file descriptor.

A unix domain socket in the local file system is specified by **unix:[name]**, where **[name]** is the name of the socket file

A UDP or (connectionless) SCTP port is specified by **[proto]:[host]:[port]**, where **[proto]** is either "udp" or "sctp" and **[host]** is the name of the destination host and **[port]** is a UDP or SCTP port number on the destination host. Please note that the length of log messages is bounded by the minimum safe UDP packet size − log messages containing more than 548 characters will be truncated.

Finally, log messages can also be sent to a small GUI by specifying the destination **@gui@**. The GUI requires a working Perl/Tk installation and a running X server.

In order to capture all log messages concerning the command line processing this option should be specified in front of all other options.

−v, −−verbose 0..10 [default: 5]
Specify the verbosity 0, 1, ..., 10 of generated / emitted log messages. The meaning of the different log levels is as follows:

- **0 (QUIET)** do not emit log messages at all.
- **1 (ERROR)** only emit severe error messages.
- **2 (WARNING)** only report severe errors and warnings.
- **3 (NOTICE)** report all errors and warnings including important informal messages.
- **4 (INFO)** report all errors, warnings and all informal messages.
- **5 (THROW)** in case of an exception, try to give a diagnosis of the error; sometimes even gives a backtrace of the current function stack.
- **6 (DEBUG0)** emit the more important debugging messages.
- **7 (DEBUG1)** emit the less important debugging messages
- **8 (DEBUG2)** emit the superfluous debugging messages
- **9 (DEBUG3)** emit annoying debugging messages.
- **10 (DEBUG4)** don't dare to use it!

−t, −−tolerance <val> [default: 1e−9]
Specifies a constraint violation tolerance value. This parameter can be used to tolerate a certain constraint violation. Use with care.

SOLVER OPTIONS

−a, −−apsolve
Solve the individual linear equation systems of the cumomer or EMU cascade using arbitrary precision arithmetic. Note that the results are converted to double precision immediately after solution. This option eliminates any round−off for the solution of the individual network levels.

−A, −−exact
In addition to option **−a** absolutely all computations are carried out using arbitrary precision arithmetic. If this option is used in conjunction with an analytical gradient (**−g analytic**) this results in exact derivatives. The results are converted to double precision just before writing them to the output file. This option is intended for debugging purposes. For large networks models the use of arbitrary precision arithmetic is probably to expensive.

−d, −−dbgsolve
When this option is used the network model is simulated in numerical debugging mode: for every equation system condition numbers are computed and the residual of the solution is checked.

STATISTICS OPTIONS

−s, −−statistics
Compute linearized statistics for the current flux setting. This option is most beneficial if the flux setting is already optimized, i.e. results from an invocation of **fitfluxes**. Indeterminable fluxes will be reported. For all determinable fluxes standard deviations will be written to the output file (**−o**). If specified together with option **−H** (HDF5 export) the Jacobian of the measurements as well as the covariance matrix of the fluxes are exported into the HDF5 file.

−g, −−gradient <arg> [default: fd4]
The desired method for computaton of partial derivatives in case statistics are to be computed (option **−s**). Possible values are '**fd1**', '**fd2**', '**fd3**', '**fd4**' for finite difference approximations, and '**analytic**' for exact derivatives. The default value is '**fd4**', i.e. the gradient is approximated using an O(hˆ4) finite difference formula (which is slightly faster than using the exact derivatives).

EXAMPLES

[source,shell]:

multifwdsim −i model.fml −H samples.hdf5

SEE ALSO

fluxml(5), fitfluxes(5), fwdsim(1),

AUTHOR

Multifwdsim extends fwdsim(1), which was originally written by Michael Weitzel.

This manpage was written by Tolga Dalman <info@13cflux.net> and Michael Weitzel <mich@el−weitzel.de> .

NAME

multiperturb − parallel 13CFLUX2 measurement and flux value perturbator

SYNOPSIS

multipertub [*options*]

DESCRIPTION

Multiperturb is a parallel implemenetation of **perturb(1)**. Like the **perturb(1)** application, FluxML documents are taken as input along with several parameters. Unlike <perturb>perturb(1)</perturb>, the output is either a directory eventually containing the result files (i.e., FluxML files containing perturbed measurements or flux values). Alternatively, **multiperturb** emits an HDF5 file containing perturbed measurements resp. flux values.

This program is especially useful for Monte Carlo Bootstrap in combination with **multifitfluxes(1)** (see EXAMPLES section below).

COMMON OPTIONS

−h, −−help
Show a brief help for all command line options.

−i, −−in <FILE> [default: stdin]
The name of the FluxML (XML) input file. If omitted, the FluxML document is expected on standard input.

−o, −−out <PATH> [default: stdout]
Output directory for FWDSIM (XML) files.

−c, −−configure <CFG> [default: 'default']
Because FluxML documents may contain several **<configuration/>** elements this option allows to specify the configuration that should be used for the simulation. If this option is omitted it is assumed that the FluxML document contains a configuration with the name "default".

−N, −−max−procs <NUM> [default: 1]
Number of maximum parallel processes

−B, −−block−size <NUM> [default: auto]
Number of samples per task. This option controls the parallelism granularity

−l, −−log DEST
Specify the destination for the internal logging. In the most simple case **DEST** is a file name of a log file. In case the file exists new log messages are appended. Apart from log files it is possible to publish log messages to file descriptors, UNIX domain sockets, UDP and SCTP ports, and a small graphical user interface.

A file descriptor is specified by **fd:[num]**, where **[num]** is the number of the file descriptor.

A unix domain socket in the local file system is specified by **unix:[name]**, where **[name]** is the name of the socket file

A UDP or (connectionless) SCTP port is specified by **[proto]:[host]:[port]**, where **[proto]** is either "udp" or "sctp" and **[host]** is the name of the destination host and **[port]** is a UDP or SCTP port number on the destination host. Please note that the length of log messages is bounded by the minimum safe UDP packet size − log messages containing more than 548 characters will be truncated.

Finally, log messages can also be sent to a small GUI by specifying the destination **@gui@**. The GUI requires a working Perl/Tk installation and a running X server.

In order to capture all log messages concerning the command line processing this option should be specified in front of all other options.

−v, −−verbose 0..10 [default: 5]

Specify the verbosity 0, 1, ..., 10 of generated / emitted log messages. The meaning of the different log levels is as follows:

- **0 (QUIET)** do not emit log messages at all.
- **1 (ERROR)** only emit severe error messages.
- **2 (WARNING)** only report severe errors and warnings.
- **3 (NOTICE)** report all errors and warnings including important informal messages.
- **4 (INFO)** report all errors, warnings and all informal messages.
- **5 (THROW)** in case of an exception, try to give a diagnosis of the error; sometimes even gives a backtrace of the current function stack.
- **6 (DEBUG0)** emit the more important debugging messages.
- **7 (DEBUG1)** emit the less important debugging messages
- **8 (DEBUG2)** emit the superfluous debugging messages
- **9 (DEBUG3)** emit annoying debugging messages.
- **10 (DEBUG4)** don't dare to use it!

PERTURB OPTIONS

−H, −−hdf5−out <FILE>
Output HDF5 file containing flux samples.

−n, −−perturbations <NUM>
Number of total perturbations (either measurements of flux samples).

−m, −−measurements <NUM>
Perturb measurements using standard model error scaled by factor <NUM> . This is useful for tuning larger or smaller perturbation ranges of the input FluxML model.

−f, −−fluxes <NUM>
Perturb flux values with a randomly by <NUM> percent according to a uniform The flux distribution

−t, −−types <TYPE LIST>
When perturbing measurements, a list of measurement types can be specified instead of all types. 'LIST' is a comma−separated list of measurement types. Valid values are: MS, MSMS, 1HNMR, 13CNMR, GENERIC, FLUX, and POOL. See the EXAMPLES section for an elaborated example.

EXAMPLES

[source,shell]:

multiperturb −i model.fml −H output.hdf5

SEE ALSO

fluxml(5), perturb(1),mcbootstrap(1)

AUTHOR

This manpage was written by Tolga Dalman <info@13cflux.net> and Michael Weitzel <mich@el−weitzel.de>.

NAME

perturb − flux and measurement value perturbation

SYNOPSIS

perturb [*options*]

DESCRIPTION

The program **perturb** is used for perturbing flux and labeling measurement values given in a FluxML document based on normally distributed random numbers. The mean of a resulting (perturbed) measurement value is given by the original measurement value. The standard deviation used for perturbation is the (scaled) standard deviation specified along with the original measurement value. On exit, **perturb** writes a modified FluxML document containing the perturbed measurement values.

This program is useful for statistical applications, like the computation of Monte Carlo bootstrap statistics.

COMMON OPTIONS

−h, −−help
Show a brief help for all command line options.

−i, −−in <FILE> [default: stdin]
The name of the FluxML (XML) input file. If omitted, the FluxML document is expected on standard input.

−o, −−out <FILE> [default: stdout]
The name of the output FluxML file. If this option is omitted the resulting FluxML document is written to standard output.

−c, −−configure <CFG> [default: 'default']
Because FluxML documents may contain several **<configuration/>** elements this option allows to specify the configuration that should be used for the simulation. If this option is omitted it is assumed that the FluxML document contains a configuration with the name "default".

−m, −−measurements <FACTOR>
This option specifies the scaling factor for the standard deviation of the measurement values. A scaling factor of one indicated that the original standard deviation should be used (i.e. no scaling).

−f, −−fluxes <PERCENTAGE>
Perturb flux values randomly by <NUM> percent according to a uniform distribution.

−t, −−types <TYPE LIST>
This option can be used to restrict the types of measurement groups which get perturbed. TYPE LIST is a comma−separated list of measurement group types. Valid group types are: MS, MSMS, 1HNMR, 13CNMR, GENERIC, FLUX, and POOL. The default behavior is to perturb all types of measurement groups.

−l, −−log DEST
Specify the destination for the internal logging. In the most simple case **DEST** is a file name of a log file. In case the file exists new log messages are appended. Apart from log files it is possible to publish log messages to file descriptors, UNIX domain sockets, UDP and SCTP ports, and a small graphical user interface.

A file descriptor is specified by **fd:[num]**, where **[num]** is the number of the file descriptor.

A unix domain socket in the local file system is specified by **unix:[name]**, where **[name]** is the name of the socket file

A UDP or (connectionless) SCTP port is specified by **[proto]:[host]:[port]**, where **[proto]** is either "udp" or "sctp" and **[host]** is the name of the destination host and **[port]** is a UDP or SCTP port number on the destination host. Please note that the length of log messages is bounded by the minimum safe UDP packet size − log messages containing more than 548 characters will be truncated.

Finally, log messages can also be sent to a small GUI by specifying the destination **@gui@**. The GUI requires a working Perl/Tk installation and a running X server.

In order to capture all log messages concerning the command line processing this option should be specified in front of all other options.

−v, −−verbose 0..10 [default: 5]

Specify the verbosity 0, 1, ..., 10 of generated / emitted log messages. The meaning of the different log levels is as follows:

- **0 (QUIET)** do not emit log messages at all.
- **1 (ERROR)** only emit severe error messages.
- **2 (WARNING)** only report severe errors and warnings.
- **3 (NOTICE)** report all errors and warnings including important informal messages.
- **4 (INFO)** report all errors, warnings and all informal messages.
- **5 (THROW)** in case of an exception, try to give a diagnosis of the error; sometimes even gives a backtrace of the current function stack.
- **6 (DEBUG0)** emit the more important debugging messages.
- **7 (DEBUG1)** emit the less important debugging messages
- **8 (DEBUG2)** emit the superfluous debugging messages
- **9 (DEBUG3)** emit annoying debugging messages.
- **10 (DEBUG4)** don't dare to use it!

EXAMPLES

There are two principal ways of using **perturb**.

Flux values are perturbed by supplying the −f flag with a percentage value. The following example will set the fluxes perturbed by 100 percent:

perturb −i network.fml −o network_noise.fml −f 1

Measurements are perturbed with respect to the defined measurement specification in the FluxML document, that is the standard deviations of the measurement device.

perturb −i network.fml −o network_noise.fml −m 2

This command will perturb all measurements by 200 percent of the standard deviation.

With parameter −t, only defined measurement groups are perturbed. The following example only perturbs MS and MSMS measurements by 100 percent of the device's standard deviation:

perturb −i network.fml −o network_noise.fml −m 1 −t MS,MSMS

Perturb also allows defining −m and −f simultaneously, causing both measurements and flux values to be perturbed:

perturb −i network.fml −o network_noise.fml −m 1 −f 1

SEE ALSO

sscanner(1), ssampler(1), setfluxes(1)

AUTHOR

Tolga Dalman and Michael Weitzel

This manpage was written by Tolga Dalman <t.dalman@fz−juelich.de>.

NAME

setfluxes − transfer (free) flux settings from FWDSIM, HDF5, or CSV to FluxML.

SYNOPSIS

setfluxes [*options*]

DESCRIPTION

Setfluxes is a tool for transferring settings of free fluxes from FWDSIM (XML), HDF5, or CSV documents to FluxML. Most common use cases are:

1. using **fitfluxes**(1), you generated optimized flux settings which are now available in a FWDSIM (XML) file. In order to do linearized statistics with **fwdsim**(1) you use **setfluxes** for transferring the flux settings to a FluxML document.
2. using **ssampler**(1), you generated thousands of random flux distributions in order to do a multi−start optimization with **fitfluxes**(1). You can now use **setfluxes**(1) to write the flux distributions, one after the other, to a FluxML document.
3. using the spreadsheet application of your choice, you created a CSV file containing a flux distribution for your network. Instead of manually editing the FluxML fail you can use **setfluxes** to automate this task.

COMMON OPTIONS

−h, −−help
Show a brief help for all command line options.

−i, −−in <FILE> [default: stdin]
The name of the FluxML (XML) input file. If omitted, the FluxML document is expected on standard input.

−o, −−out <FILE> [default: stdout]
The name of the FluxML output file. If omitted, the modified FluxML document is written to standard output.

−c, −−configure <CFG> [default: 'default']
Because FluxML documents may contain several **<configuration/>** elements this option allows to specify the configuration that should be used for the simulation. If this option is omitted it is assumed that the FluxML document contains a configuration with the name "default".

DATA SOURCE OPTIONS

−F, −−fwd <FILE>
Specify a FWDSIM (XML) file as data source, usually generated by either **fwdsim**(1) or **fitfluxes**. The layout of these files is documented in manual page **fwdsim**(5).

−H, −−hdf <FILE>
Use a HDF5 file as data source. The HDF5 file is expected to have the following layout:

- '/flux/names' should be a vector of strings containing the flux names with suffixes '.n' or '.x' for net and exchange fluxes
- '/flux/data' should be a matrix of double precision floating point numbers. The columns of the matrix are associated with the flux names in the elements of '/flux/names'

In case the line number (option **−l**) is omitted it is assumed that the first dataset in line 1 should be transferred.

−C, −−csv <FILE>
Use this parameter to specify a CSV file containing the flux distribution. The CSV file should have the following layout:

- a column heading (the first row) should list the flux names with suffixes '.n' or '.x' for net and exchange fluxes

- starting with the second row each row of the CSV file contains one complete flux setting

In case the line number (option **−l**) is omitted it is assumed that the first dataset in line 1 (row 2) should be transferred.

−l, −−line <LINENUM> [default: 1]
When using a HDF5 or CSV file as data source, specify the line number (row number) of the flux distribution which should be written to the generated FluxML document. If this parameter is omitted the first first flux distribution is selected.

SPECIAL OPTIONS

−f, −−force
Write all flux settings to the generated FluxML document without checking whether they existed in the input FluxML document. Use this option if your input FluxML document contained no settings for the free fluxes, or the choice of free fluxes in the input FluxML file does not match the choice in the data source. If this parameter is not specified **setfluxes** tries to detect non−matching combinations of data source and FluxML input document.

−D, −−data <PATH> [default: '/flux/data']
For a HDF5 data source, specify the path to the flux values. Use with care.

−N, −−names <PATH> [default: '/flux/names']
For a HDF5 data source, specify the path to the flux names. Use with care.

EXAMPLES

Transfer the flux distribution found in a FWDSIM document to a corresponding FluxML document network.fml (most simple case):

setfluxes −i network.fml −F network.fwd −o network_new.fml

Transfer the 123'rd flux distribution found in a HDF5 file samples.h5 to a corresponding FluxML document network.fml:

setfluxes −i network.fml −H samples.h5 −l 123 −o network_new.fml

Convert an old FTBL network specification to FluxML and replace the flux settings by the values contained in line 5 of a CSV file. Simulate the result using **fwdsim**(1):

ftbl2fml −i network.ftbl | setfluxes −C samples.csv −l 5 | fwdsim −o results.fwd

SEE ALSO

fwdsim(1), fitfluxes(1), ftbl2fml(1)

AUTHOR

Michael Weitzel and Tolga Dalman

This manpage was written by Michael Weitzel <mich@el−weitzel.de>.

NAME

setmeasurements − Transferring labeling and flux measurements from FWDSIM, FluxML or CSV to FluxML.

SYNOPSIS

setmeasurements [*options*]

DESCRIPTION

setmeasurements is a tool for transferring labeling or flux measurements from FWDSIM, FluxML or CSV documents to FluxML. Most common use cases are:

1. using **fwdsim** with simulation type full to generate a set of labeling and flux measurements in a FWDSIM file.
2. building up defined flux and labeling measurements in a CSV file to import them into a FluxML file.
3. copying a set of measurement data from an existing FluxML file to a new FluxML file without data.

COMMON OPTIONS

−h, −−help
Show a brief help for all command line options.

−i, −−in <FILE> [default: stdin]
The name of the FluxML (XML) input file. If omitted, the FluxML document is expected on standard input.

−o, −−output <FILE> [default: stdout]
The name of the FluxML output file. If omitted, the modified FluxML document is written to standard output.

−c, −−configure <CFG> [default: 'default']
Because FluxML documents may contain several **<configuration/>** elements this option allows to specify the configuration that should be used for the simulation. If this option is omitted it is assumed that the FluxML document contains a configuration with the name "default".

DATA SOURCE OPTIONS

−F, −−fwd <FILE>
Specify a FWDSIM (XML) file as data source, usually generated by either **fwdsim**(1) or **fitfluxes**. The layout of these files is documented in manual page **fwdsim**(5).

−X, −−fml <FILE>
Use a FluxML file as data source.

−C, −−csv <FILE>
Use this parameter to specify a CSV file containing the flux and labeling measurements. The CSV file should have the following layout:

Table 1. Format of CSV labeling and flux measurements

#specifications	measured values	measured stddevs
net:co2_exp	4.147718258	0.538999605
"PGA#M0,1,2,3"	0.54637224	0.007434449
	0.129020493	0.001872506
	0.070576643	0.001663439
	0.254030624	0.004767783

The first column contains the specifications of the used flux or labeling measurement. Fluxes has to be marked with "net:" for automated detection. In the second column the measured value of the flux or the labeling fraction is stored with the corresponding standard deviation in the third column. The specifications of the used measurement groups is denoted in the .xsd scheme at

www.13cflux.net/fluxml. The CSV file should use comma as separator. All values without a specification are added to the previous analyte.

−f, −−filter_flux <REGEX>
Only output the data sets which labeling measurement group name is matched by the specified regular expression. If omitted all measurement groups are imported. Only in combination with −F. See regex(7) for the syntax of regular expressions.

−l, −−filter_label <REGEX>
Only output the data sets which flux measurement name is matched by the specified regular expression. If omitted, none of the flux measurements is imported. Only in combination with −F. See regex(7) for the syntax of regular expressions.

SPECIAL OPTIONS

−R, −−num <NUMBER> [default: 0.05]
Relative standard deviation for measurement data from FWDSIM files. Only in combination with −F.

−A, −−num <NUMBER> [default: 0.01']
Absolute standard deviation for measurement data from FWDSIM files. Only in combination with −F.

EXAMPLES

Transfer all labeling measurements found in a FWDSIM document to a corresponding FluxML document network.fml (most simple case):

setmeasurements −i network.fml −F network.fwd −o network_new.fml

As above with specific absolute and relative deviations:

setmeasurements −i network.fml −F network.fwd −o network_new.fml −R 0.1 −A 0.02

Import labeling and flux measurements from an CSV file to a FluxML document network.fml:

setmeasurements −i network.fml −C measurement_data.csv −o network_new.fml

Transfer all labeling measurements found in a FluxML document to a corresponding FluxML document network.fml:

setmeasurements −i network.fml −X network2.fml −o network_new.fml

SEE ALSO

setfluxes(1), fwdsim(1), fitfluxes(1), ftbl2fml(1)

AUTHOR

Stephan Miebach

This manpage was written by Stephan Miebach <info@13cflux.net> and Michael Weitzel <mich@el−weitzel.de>.

NAME

simreport − simulation report generator.

SYNOPSIS

simreport [*options*]

DESCRIPTION

Given a FluxML file and a corresponding simulation results in form of a FWDSIM (XML) file **simreport** generates a CSV file comparing the given and simulated measurement values. Norm values, measuring the discrepancy between measurement and simulation are provided for all measurement values.

OPTIONS

−h, −−help
Show a brief help for all command line options.

−i, −−fwdsim <FILE> [default: stdin]
The name of the FWDSIM (XML) input document. Default is to read from standard input.

−f, −−fluxml <FILE>
The name of the FluML input file (optional). In case the name of the FluxML file is omitted simreport generates just a listing of the simulated measurement values.

−o, −−out <FILE> [default: stdout]
Name of the generated CSV file. If this option is not specified the generated CSV file is written to standard output.

−c, −−configure <CFG> [default: 'default']
Because FluxML documents may contain several **<configuration/>** elements this option allows to specify the configuration that should be used for the simulation. If this option is omitted it is assumed that the FluxML document contains a configuration with the name "default".

EXAMPLES

Generate a CSV simulation report file from a FluxML document and a FWDSIM (XML) document (most simple case):

simreport −i network.fwd −f network.fml −o report.csv

Combine **simreport** with **fwdsim**, i.e. read the FWDSIM (XML) document from standard input:

fwdsim −i network.fml | simreport −f network.fml −o report.csv

Produce a nice representation of the simulated measurement values. The generated CSV file will not contain the real measurement values, norms, or group scaling factors:

fwdsim −i network.fml | simreport −o report.csv

SEE ALSO

fwdsim(1), fitfluxes(1), fwdsimflt(1)

AUTHOR

Michael Weitzel

This manpage was written by Michael Weitzel <mich@el−weitzel.de>.

NAME

sscanner − generate an initial flux distribution for a metabolic network

SYNOPSIS

sscanner [*options*]

DESCRIPTION

The program **sscanner** is used to generate an initial flux distribution for a metabolic network. In order to perform this task **sscanner** reads a FluxML document (see also **fluxml**(1)), chooses some free fluxes and reinitializes the flux values with the analytical center of the specified constraints.

COMMON OPTIONS

−h, −−help
Show a brief help for all command line options.

−i, −−in <FILE> [default: stdin]
The name of the FluxML (XML) input file. If omitted, the FluxML document is expected on standard input.

−o, −−out <FILE> [default: stdout]
The name of the output FluxML file. If this option is omitted the resulting FluxML document is written to standard output.

−c, −−configure <CFG> [default: 'default']
Because FluxML documents may contain several **<configuration/>** elements this option allows to specify the configuration that should be used for the simulation. If this option is omitted it is assumed that the FluxML document contains a configuration with the name "default".

−L, −−list
Specifying this option results in a list of allowed configuration names for the specified FluxML document. The program exits immediately after emitting the list.

−l, −−log DEST
Specify the destination for the internal logging. In the most simple case **DEST** is a file name of a log file. In case the file exists new log messages are appended. Apart from log files it is possible to publish log messages to file descriptors, UNIX domain sockets, UDP and SCTP ports, and a small graphical user interface.

A file descriptor is specified by **fd:[num]**, where **[num]** is the number of the file descriptor.

A unix domain socket in the local file system is specified by **unix:[name]**, where **[name]** is the name of the socket file

A UDP or (connectionless) SCTP port is specified by **[proto]:[host]:[port]**, where **[proto]** is either "udp" or "sctp" and **[host]** is the name of the destination host and **[port]** is a UDP or SCTP port number on the destination host. Please note that the length of log messages is bounded by the minimum safe UDP packet size − log messages containing more than 548 characters will be truncated.

Finally, log messages can also be sent to a small GUI by specifying the destination **@gui@**. The GUI requires a working Perl/Tk installation and a running X server.

In order to capture all log messages concerning the command line processing this option should be specified in front of all other options.

−v, −−verbose 0..10 [default: 5]
Specify the verbosity 0, 1, ..., 10 of generated / emitted log messages. The meaning of the different log levels is as follows:

- **0 (QUIET)** do not emit log messages at all.

- **1 (ERROR)** only emit severe error messages.
- **2 (WARNING)** only report severe errors and warnings.
- **3 (NOTICE)** report all errors and warnings including important informal messages.
- **4 (INFO)** report all errors, warnings and all informal messages.
- **5 (THROW)** in case of an exception, try to give a diagnosis of the error; sometimes even gives a backtrace of the current function stack.
- **6 (DEBUG0)** emit the more important debugging messages.
- **7 (DEBUG1)** emit the less important debugging messages
- **8 (DEBUG2)** emit the superfluous debugging messages
- **9 (DEBUG3)** emit annoying debugging messages.
- **10 (DEBUG4)** don't dare to use it!

SPECIAL OPTIONS

−B, −−bounds
Determine the lower and upper bounds for the settings of the free fluxes by using an LP solver. The program exits immediately after emitting the list.

−C, −−constraints
This option causes a list of user defined and automatically inferred equality and inequality constraints to be generated. The program exits immediately after emitting the list.

−m, −−montecarlo <NUM>
Determine the setting of the free fluxes from the centroid of point cloud generated using Gibbs sampling, a Markov Chain Monte Carlo technique. This parameter requires the number of Monte Carlo samples to be specified.

−r, −−resample <n,x,nx> [default: nx]
This option is used to specify what to resample. Possible values are 'n', 'x', and 'nx' − for sampling only net fluxes, only exchange fluxes, or both net and exchange fluxes. The default is to resample both net and exchange fluxes.

−b, −−bound−net <VALUE> [default: 100]
The bound for the magnitude of unbounded net fluxes. Defaults to 100.

−p, −−bound−xch <VALUE> [default: 100]
The bound for unbounded exchange fluxes. Defaults to 100.

EXAMPLES

Generate a new flux distribution for the network in FluxML file network.fml representing the analytical center of the specified stoichiometric constraints. In case the stoichiometry of network.fml is unbounded a default bound of 100 is used for all unbounded net and exchange fluxes. The resulting flux distribution is written to another FluxML file.

sscanner −i network.fml −o new_network.fml

The same as above, however, the free fluxes are initialized to represent the centroid of a cloud of 100 uniformly distributed points. If 1 instead of 100 points are used this allows a random initialization of the stoichiometry (see also ssampler(1)):

sscanner −m 100 −i network.fml −o new_network.fml

Compute the lower and upper bounds of the free net and exchange fluxes. The result is written to standard output:

sscanner −i network.fml −B

SEE ALSO

fwdsim(1), fitfluxes(1), ssampler(1)

AUTHOR

Michael Weitzel

This manpage was written by Michael Weitzel <mich@el−weitzel.de>.

NAME

ssampler − sample random flux distributions from the stoichiometry of a metabolic network.

SYNOPSIS

ssampler [*options*] [−o <FILE>]

DESCRIPTION

Ssampler (stoichiometry sampler) is a tool for sampling uniformly distributed, random flux distributions from the stoichiometry of a metabolic reaction network. The specification of the reaction network is assumed to be contained in a FluxML document. The generated flux distributions are saved to a HDF5 file. **Ssampler** is used to generate initial feasible flux distributions and random samples for distributed multi−start optimization.

COMMON OPTIONS

−h, −−help
Show a brief help for all command line options.

−i, −−in <FILE> [default: stdin]
The name of the FluxML (XML) input file. If omitted, the FluxML document is expected on standard input.

−o, −−out <FILE>
The name of the HDF5 output file. This option is required (HDF5 files are never written to standard output).

−c, −−configure <CFG> [default: 'default']
Because FluxML documents may contain several **<configuration/>** elements this option allows to specify the configuration that should be used for the simulation. If this option is omitted it is assumed that the FluxML document contains a configuration with the name "default".

−L, −−list
Specifying this option results in a list of allowed configuration names for the specified FluxML document. The program exits immediately after emitting the list.

−l, −−log DEST
Specify the destination for the internal logging. In the most simple case **DEST** is a file name of a log file. In case the file exists new log messages are appended. Apart from log files it is possible to publish log messages to file descriptors, UNIX domain sockets, UDP and SCTP ports, and a small graphical user interface.

A file descriptor is specified by **fd:[num]**, where **[num]** is the number of the file descriptor.

A unix domain socket in the local file system is specified by **unix:[name]**, where **[name]** is the name of the socket file

A UDP or (connectionless) SCTP port is specified by **[proto]:[host]:[port]**, where **[proto]** is either "udp" or "sctp" and **[host]** is the name of the destination host and **[port]** is a UDP or SCTP port number on the destination host. Please note that the length of log messages is bounded by the minimum safe UDP packet size − log messages containing more than 548 characters will be truncated.

Finally, log messages can also be sent to a small GUI by specifying the destination **@gui@**. The GUI requires a working Perl/Tk installation and a running X server.

In order to capture all log messages concerning the command line processing this option should be specified in front of all other options.

−v, −−verbose 0..10 [default: 5]
Specify the verbosity 0, 1, ..., 10 of generated / emitted log messages. The meaning of the different log levels is as follows:

- **0 (QUIET)** do not emit log messages at all.
- **1 (ERROR)** only emit severe error messages.
- **2 (WARNING)** only report severe errors and warnings.
- **3 (NOTICE)** report all errors and warnings including important informal messages.
- **4 (INFO)** report all errors, warnings and all informal messages.
- **5 (THROW)** in case of an exception, try to give a diagnosis of the error; sometimes even gives a backtrace of the current function stack.
- **6 (DEBUG0)** emit the more important debugging messages.
- **7 (DEBUG1)** emit the less important debugging messages
- **8 (DEBUG2)** emit the superfluous debugging messages
- **9 (DEBUG3)** emit annoying debugging messages.
- **10 (DEBUG4)** don't dare to use it!

SAMPLING OPTIONS

−n, −−nsamples <NUM> [default: 1000]
The number of samples to be generated. Defaults to 1000 if omitted.

−s, −−sample <n,x,nx> [default: n]
This option is used to specify what to sample. Possible values are 'n', 'x', and 'nx' − for sampling only net fluxes, only exchange fluxes, or both net and exchange fluxes. The default is to sample only the distribution of net fluxes.

−S, −−sampler <g,h> [default: g]
Use this parameter to specify the sampling method. The default sampling method is Gibbs sampling ('g') which usually gives the best results. Alternatively, Hit−And−Run sampling ('h') may be used (slightly faster).

−b, −−bound−net <VALUE> [default: 100]
The bound for the magnitude of unbounded net fluxes. Defaults to 100.

−p, −−bound−xch <VALUE> [default: 100]
The bound for unbounded exchange fluxes. Defaults to 100.

−f, −−faster
Especially if the metabolic network is very large, the sampling process may be very slow. In this case the user has to option to skip the warmup phase performed after each step of the sampler. This results in much faster sampling. However, the generated samples may be of lower quality, i.e. less uniformly distributed.

−r, −−randomize <NUM> [default: 0]
This option allows to specify a randomization interval. After the specified number of steps the sampler is re−initialized with a randomized start point. Specify a value greater than 0 if you have the feeling that the generated random samples are not uniformly distributed, which is sometimes the case for a degenerate flux space. This option defaults to 0, i.e. randomization is disabled.

EXAMPLES

Sample a random net flux distribution consisting of 1000 random samples using the Gibbs sampling method (most simple case):

ssampler −i network.fml −o samples.h5

Sample net and exchange fluxes and generate 10 flux distributions. Bound the net fluxes to +/−15.5 and use the Hit−And−Run sampling method:

ssampler −i network.fml −o samples.h5 −n 10 −p 15.5 −S h −o network_new.fml

SEE ALSO

fwdsim(1), fitfluxes(1), sscanner(1)

AUTHOR

Michael Weitzel

This manpage was written by Michael Weitzel <mich@el−weitzel.de>.

Appendix

13CFLUX2 – a short reference guide

For a gentle introduction and overview over the genera methodology of modeling, simulation, design and statistical analysis of ^{13}C-labeling experiments for metabolic flux analysis (short 13C-MFA) we refer to Wiechert et al. *A Universal Framework for ^{13}C Metabolic Flux Analysis*, Metab Eng 3, 265-83, 2001, doi:10.1006_mben.2001.0188.

Category	13CFLUX2 program (page)	Command line call	Input	Output	Additional options/ annotations	General comments
**Help and Online Documentation**						
	any program	program - h		syntax and command line options of the program		provides list of command line options
		man program		manual page of the program		additionally provides example program calls and special options
		program -v num<br>program –verbose num	number = 0..10			specifies the program's degree of verbosity
		program > output.txt		text file output.txt		redirects program output on stdout to a file
**Simulation and Parameter Fitting**						
	fwdsim<br>(26)	fmllint -i input.fml	FluxML file input.fml			forward simulation and statistical analysis: *the* core operation of automatic equation generation and numerical solution of label balances from a given feasible 13C-MFA model (FluxML file). fwdsim simulates the distribution of labeled material all over the network.
		fmllint -i input.fml -o output.fwd		FWDSIM file output.fwd containing fluxes and simulated measurements		
		fwdsim -i input.fml -o output.fwd -s		FWDSIM file output.fwd containing simulated measurements and fluxes along with their standard deviations		Structural and statistical identifiability analysis: confidence regions of fluxes for given measurement specification according to linearized statistics; structurally non-identifiable

						fluxes should be fixed (excluded from parameter estimation); flux confidence regions depend on input label mixture, measurements and their standard deviations
		fwdsim -i input.fml -o output.fwd -H output.hdf5<br><br>fwdsim -i input.fml -o output.fwd -M output.mml<br><br>fwdsim -i input.fml -o output.fwd -T output.txt		HDF5-/ MathML-/TXT-file output.hdf5/ output.mml/ output.txt containing stoichiometry and cascaded systems		provides information for rapid application development with MATLAB or Computer Algebra systems
		fwdsim -i input.fml -o output.fwd -H output.hdf5 -s		HDF5 file output.hdf5 containing the Jacobian and covariance matrices		provides additional matrices to be used for model debugging (e.g. to calculate parameter and output sensitivities)
	multifwdsim (43)	multifwdsim -i input.fml -o output_folder -H samples.hdf5	FluxML file input.fml; HDF5 file samples.hdf5 containing random feasible flux distributions (generated by ssampler)	directory output_folder for the generated FWDSIM files with fluxes and simulated measurements		parallel implementation of fwdsim for variation studies and sensitivity analyses. As for fwdsim, FluxML documents are taken as input along with several parameters. The output is a directory containing the FWDSIM files containing simulation results.
		multifwdsim -i input.fml -o output_folder -H samples.hdf5 -s		directory output_folder for the output FWDSIM files with all simulated labeling measurements and fluxes along with their standard deviations		
	fitfluxes (17)	fifluxes -i input.fml -o output.fwd	FluxML file input.fml	FWDSIM file output.fwd containing optimized fluxes and simulated measurements		parameter estimation: fitfluxes intelligently adjusts a set of flux values for a metabolic network to reproduce a set of isotope labeling measurement values as good as possible. Always start fitfluxes from different sets of initial flux

						values to account for the local-global optima issue of nonlinear optimization
		fitfluxes -i input.fml -o output.fwd<br>-O NAGNLP (IPOPT)			-O:<br>specifies optimization routine to be used; select the commercial optimizer NAGNLP if licensed or the free optimizer IPOPT<br>[default: IPOPT]	for some cases NAGNLP is found to be faster and more robust then IPOPT
		fitfluxes -i input.fml -o output.fwd<br>-O NAGNLP<br>-P "nag_opt_nlp.max_iter=integer(50)"			-P:<br>comma separated list of optimizer options; example: restriction of the maximum number of iterations to 50<br>[default: 1000]	
	multifitfluxes<br>(39)	multifitfluxes -i input.fml<br>-H samples.hdf5<br>-o output_folder	FluxML file input.fml;<br>HDF5 file samples.hdf5 containing feasible random flux distributions for input.fml (generated by ssampler)	directory output_folder for the output FWDSIM files containing optimized fluxes and simulated measurements<br>(the number of FWDSIM files depends on the number of performed parameter fittings;<br>the name of the output files is given by the input file name with increasing number)	In contrast to multifit an output folder (or output HDF5) has to be specified and flux distributions (generated by ssampler) have to be provided.	multifitfluxes is a parallel implementation of fitfluxes (and newer variant of multifit).<br>The output is either a directory containing the result files (i.e., FWDSIM files containing parameter estimation results), or a unified HDF5 file comprising all best fit flux results.
		multifitfluxes -i input.fml<br>-H samples.hdf5 -f output.hdf5		HDF5 file output.hdf5 containing all optimized fluxes values		
		multifitfluxes -i input.fml<br>-o output_folder -H samples.hdf5<br>-O NAGNLP<br>-P "nag_opt_nlp.max_iter=integer(50)"			-O and –P:<br>optimizer parameters to be used as in fitfluxes	
	multifit<br>(obsolete)<br>(37)	multifit -i input.fml -n 10	FluxML file input.fml	the names of the output files<br>output.fml_NUM.fwd are given by the input file name and an increasing number NUM;<br>FWDSIM files contain optimized fluxes and	-n:<br>number of random flux distributions (generated with ssampler)	multifit is a multi-processor, multi-start optimization using fitfluxes based on random flux distributions generated by ssampler.<br>multifit performs a simple type of load balancing in order to use the available computational resource as

				simulated measurements (the number of FWDSIM files depends on the number of performed parameter fittings)		efficient as possible. Deprecated version, use multifitfluxes instead
		multifit -i input.fml -n 10<br>-o output_folder<br>-p output_name		directory output_folder for FWDSIM output files	-p: name of the output files	
		multifit -i input.fml<br>-o output_folder<br>-f samples.hdf5	HDF5 file samples.hdf5 containing feasible random flux distributions for input.fml (generated by ssampler)		with -f random flux distributions contained in samples.hdf5 (generated by ssampler) are used as initial flux values	
	mcbootstrap<br>(35)	mcbootstrap -i input.fml<br>-o ouput_folder	FluxML file input.fml	directory output_folder for the resulting FWDSIM files with all optimized fluxes and simulated measurements		mcbootstrap implements a Monte Carlo bootstrap algorithm for nonlinear statistical assessment of flux confidence regions
		mcbootstrap -i input.fml<br>-o ouput_folder<br>-n 2		p1_1.fwd p1_2.fwd	-n:<br>determines the number of created random flux distributions<br>[default: 1]	
		mcbootstrap -i input.fml<br>-o ouput_folder<br>-n 2<br>-m 3		directory output_folder containing the files<br>p1_1.fwd<br>p1_2.fwd<br>p2_1.fwd p2_2.fwd<br>p3_1.fwd p3_2.fwd	-m:<br>specifies the number of executed perturbations<br>[default: 1]	
**Sampling and Flux Initialization**						
	sscanner<br>(55)	sscanner -i input.fml -o output.fml	FluxML file input.fml	FluxML file output.fml: modified input FluxML file containing feasible flux samples		sscanner generates an initial feasible flux distribution for a metabolic network; free fluxes are suggested if not given resp. checked for validity if specified; flux values are re-initialized with the analytical center of the specified flux constraints

						(i.e., center of the stoichiometric flux space)
		sscanner -i input.fml -o output.fml<br>-r nx			-r<br>sampling targets: net fluxes [n], xch fluxes [x], or both [nx]<br>[default: n]	
		sscanner -i input.fml -o output.fml<br>-b 1.5			-b upper bound for net fluxes<br>[default: 100]	
		ssampler -i input.fml -o output.fml<br>-r nx<br>-p 1000			-p upper bound for exchange fluxes<br>[default: 100]	
	ssampler<br>(58)	ssampler -i input.fml<br>-o output.hdf5<br>-n 10	FluxML file input.fml	HDF5 file output.hdf5 containing feasible flux samples	-n:<br>number of samples to be generated<br>[default 1000]	samples uniformly distributed random feasible flux values accounting for stoichiometry of the metabolic network; to be used for multi-start "global" optimization
		ssampler -i input.fml -o output.hdf5<br>-s nx			-s:<br>sampling targets: net fluxes [n], xch fluxes [x], or both [nx]<br>[default: n]	
		ssampler -i network.fml<br>-o output.hdf5<br>-S h			-S:<br>choice of sampling method Gibbs ('g') or Hit-and-Run ('h') [default g]	
		ssampler -i input.fml -o output.hdf5<br>-b 1.5			-b:<br>upper bound for net fluxes<br>[default: 100]	
		ssampler -i input.fml -o output.hdf5<br>-s nx<br>-p 1000			-p:<br>upper bound for exchange fluxes<br>[default: 100]	
	perturb<br>(48)	perturb -i input.fml -o output.fml<br>-m 1	FluxML file input.fml	FluxML file output.fml: modified input.fml containing the perturbed measurement values	-m:<br>specifies the scaling factor for the standard deviation of the measurement values. A scaling factor of one indicates that the original standard deviation should be used (i.e., no scaling).	perturbing flux and labeling measurements given in a FluxML document based on normal probability distribution. The mean of a resulting (perturbed) measurement value is given by the original measurement value. The

						standard deviation used for perturbation is the (scaled) standard deviation specified along with the original measurement value; for nonlinear statistical analysis and global flux sensitivity analysis
		perturb -i input.fml -o output.fml<br>-m 1<br>-t MS,MSMS			-t:<br>only measurement types in <list> (comma-separated string) are perturbed. Available measurement types are: MS, MSMS, 1HNMR, 13CNMR, GENERIC, and FLUX [default: all measurement types]	
	multiperturb<br>(46)	multiperturb -i input.fml -o output_folder<br>-m 1	FluxML file input.fml	directory output_folder for containing the resulting FluxML files	-m:<br>specifies the scaling factor for the standard deviation of the measurement values. A scaling factor of one indicated that the original standard deviation should be used (i.e., no scaling)	multiperturb is a parallel implementation of perturb. Like the original perturb application, FluxML documents are taken as input along with several parameters.
		multiperturb -i input.fml -o output.fml<br>-m 1<br>-t MS,MSMS			-t:<br>only measurement types in <list> (comma-separated string) are perturbed. Available measurement types are: MS, MSMS, 1HNMR, 13CNMR, GENERIC, and FLUX [default: all measurement types]	
**Experimental Design**						
	edscanner<br>(13)	edscanner -i input.fml -m mixture.mix<br>-o output.hdf5<br>-n 500	FluxML file input.fml;<br>XML file mixture.mix containing the substrate specification	HDF5 file output.hdf5 containing coordinates of the sampling grid points and information measure	-n:<br>number of samples to be generated [defaults: 10]	edscanner samples the mixing simplex given by a mixture of specified labeling substrates at evenly spaced grid points. At each grid point a linearized statistical analysis is performed and an information measure is

						calculated.<br>In case of an input triple a mixture triangle may be visualized using the provided MATLAB script drawMixingTriangle.m.<br>Be aware that the experimental design requires an error model for all measurements providing an extrapolation of standard errors.
		edscanner -i input.fml -m mixture.mix<br>-o output.hdf5<br>-n 500<br>-A			-C:<br>Used information measure; available measures rely on A-, D-, E, and M-criteria	
	edopt<br>(8)	edopt -i input.fml -m mixture.mix<br>-o output.fml	FluxML file input.fml;<br>XML file mixture.mix containing the substrate specification	FluxML file output.fml containing optimized input substrate mixture		edopt calculates an optimal experimental design by adjusting the proportions for the specified labeling substrates to maximize information (minimize flux uncertainty) relying on linearized statistical analysis. Be aware that the experimental design requires an error model for all measurements providing an extrapolation of standard errors.
		edopt -i input.fml -m mixture.mix<br>-o output.fml<br>-C M			-C:<br>Used information measure; available measures rely on A-, D-, E, and M-criteria	
		edopt -i input.fml -m mixture.mix<br>-o output.fml<br>–O SUBPLEX			-O:<br>specifies optimization routine to be used; select the commercial optimizers NAGNLP if licensed or the free optimizers SUBPLEX, IPOPT<br>[default: IPOPT]	
		edopt -i input.fml -m mixture.mix<br>-o output.fml<br>–O NAGNLP<br>-r 50			-r:<br>number of randomly initialized multi-start optimization runs	

					[default: 1]	
**Report Generation/ (Post-)Processing**						
	fwdsimflt<br>(32)	fwdsimflt -i input.fwd -m	FWDSIM file input.fwd		-m: filters out simulated measurement values	fwdsimflt filters FWDSIM documents generated by fwdsim and fitfluxes to human-readable CSV formats; for fast screening of simulative results
		fwdsimflt -i input.fwd -s		written to stdout	-s: filters out flux names and values	
		fwdsimflt -i input.fwd -S			-S. filters out the network stoichiometry	
		fwdsimflt -i input.fwd -t			-t: filters out the measurements' standard deviations	
	simreport<br>(54)	simreport -i input.fwd -f input.fml<br>-o report.csv	FWDSIM file input.fwd containing simulated measurements	CSV file report.csv containing analysis results	-f:<br>FluxML file input.fml containing specific measurements	simreport provides a comparison between given and simulated measurement values. Norm values, measuring the discrepancy between measurement and simulation are provided; useful to determine largest contributions to the sum of squared residuals; for fast screening of simulative results
	collectfitdata<br>(6)	collectfitdata –f fwdsim_input_dir<br>–o output.hdf5	path to directory fwdsim_input_dir containing FWDSIM files	HDF5 file output.hdf5		collectfitdata gathers simulation results from multiple FWDSIM files into a single HDF5 file; helper function to build automated post-processing workflows
		collectfitdata –f fwdsim_input_dir<br>–oa output.hdf5			-a: appends information to an existing HDF5 file, otherwise the HDF5 file is created	
		collectfitdata –f fwdsim_input_dir<br>–omFX output.hdf5			-m,-F,-N,-X:<br>control options for specifying information depth to be saved (m – measurements, F – free fluxes only, N – no net	

					fluxes, X – no exchange fluxes)	
	fwdsim2csv (31)	fwdsim2csv –af *.fwd	-f: column containing FWDSIM file names	written to stdout	-a: all fluxes [default: free fluxes]	collapses flux settings from multiple FWDSIM documents into one single CSV file; helper function to build automated post-processing workflows
		fwdsim2csv –anf *.fwd			-n: output only net fluxes	
		fwdsim2csv –axf *.fwd			-x: output only exchange fluxes	
	hdf52csv (34)	hdf5tocsv –i input.hdf5 –o output.csv	HDF5 file input.hdf5	CSV file output.csv		converts an XML file to a CSV formatted output; helper function for post-processing of simulation results
		hdf5tocsv –i input.hdf5 –o output.csv -d ;			-d: specify separator character for the output CSV file [default: ,]	
**Data Transfer**						
	ftbl2fml (23)	ftbl2fml -i input.fml -o output.fml	FTBL file input.ftbl	FluxML file output.fml		converts an "old" 13CFLUX model file into a new FluxML document
	setfluxes (50)	setfluxes -i input.fml -F fluxinput.fwd -o output.fml	FluxML file input.fml; FWDSIM file fluxinput.fwdas flux source	FluxML file output.fml: modified FluxML input file containing the reset flux values		setfluxes transfers (free) flux values from several file formats FWDSIM, HDF5, or CSV into a FluxML file; helper function to build automated processing workflows
		setfluxes -i input.fml -H fluxinput.hdf5 -o output.fml -l 3	HDF5 file fluxinput.hdfas flux source		-l: specifies the line number (row) of the flux distribution to be written to output.fml [default: 1]	
		setfluxes -i input.fml -C fluxinput.csv -o output.fml -l 3	CSV file fluxinput.csvas flux source			
	setmeasurements (52)	setmeasurements -i input.fml -F measinput.fwd -o output.fml	FluxML file input.fml; FWDSIM file	FluxML file output.fml: modified FluxML input file containing the reset		setmeasurements transfers measurement values from several

			measinput.fwd as measurement source	measured values		file formats FWDSIM, HDF5, or CSV into a FluxML file; helper function to build automated processing workflows
		setmeasurements -i input.fml<br>-H measinput.hdf5<br>-o output.fml	HDF5 file measinput.hdf as measurement source			
		setmeasurements -i input.fml<br>-C measinput.csv<br>-o output.fml	CSV file fluxinput.csvas measurement source			
**Validation and Benchmark**						
	fmllint<br>(21)	fmllint -i input.fml	FluxML file input.fml	stdout: error messages/ warnings	in case the command line is empty, the FluxML document is expected	fmllint validates a FluxML file.<br>In addition to the syntactic check provided by the underlying Xerces-C XML Schema validation, fmllint performs a thorough semantic check of a FluxML document.
	benchmark<br>(4)	benchmark -i input.fml	FluxML file input.fml	stdout: benchmark/ analysis results		provides various performance benchmark measures for the 13CFLUX 2 software<br>and performs an analysis of the computational cumomer/ EMU graphs;<br>program to generate performance measurements of 13CFLUX2 (number crunching);<br>HAVE FUN ☺

ACADEMIC 13CFLUX2 SOFTWARE LICENSE AGREEMENT

AGREEMENT

BETWEEN

Forschungszentrum Jülich GmbH

52425 Jülich

Federal Republic of GERMANY

- hereinafter referred to as “Forschungszentrum Jülich” or “PUBLISHER”

AND

Institute

Department

Street Address

City

State/ Province

ZIP/ Postal Code

Country

represented by

Family name

First name

Email

- hereinafter referred to as “LICENSEE”
- hereinafter also referred to as “PARTY” and/ or “PARTIES”

CONCERNING

Use of the Software Suite **13CFLUX2**

Please read through carefully

PRAEAMBLE:

Within the Institute of Bio- and Geosciences 1 (IBG-1): Biotechnology, Modeling and Simulation Group, affiliated to the Forschungszentrum Jülich GmbH, the author Michael Weitzel and co-authors have developed the software suite **13CFLUX2**. Forschungszentrum Jülich holds all intellectual rights, titles and interests in **13CFLUX2** and is interested to have other research groups and educational institutes to also use the software.

The LICENSEE is strongly interested in obtaining a license for using the **13CFLUX2** software from Forschungszentrum Jülich for **non-commercial and/ or educational purposes**. On the assumption of a non-commercial use of **13CFLUX2** Forschungszentrum Jülich is pleased to give you the right to use the software submitting the following conditions:

1. Description of the Software

The "PUBLISHER" has designed and developed **13CFLUX2** which consists of a set of applications for performing ^{13}C-based Metabolic Flux Analysis (^{13}C-MFA), i.e. the simulation-based estimation of *in-vivo* metabolic reaction rates. In particular, the implemented type of ^{13}C-MFA relies on ^{13}C labeling measurement data obtained from an organism which is kept under metabolic and isotopic stationary conditions. The ^{13}C labeling measurement data are usually (but not necessarily) generated using mass spectrometry (MS) or nuclear magnetic resonance (NMR) measurement devices.

The estimation of *in-vivo* reaction rates for very large metabolic networks, the evaluation of a series of high throughput experiments, as well as the evaluation of nonlinear statistics and experimental design involves a high computational effort. By implementing newly developed simulation algorithms, which are highly tuned for the underlying mathematical problems, using **13CFLUX2** allows to perform ^{13}C-MFA studies on a common desktop computer. Network models and measurements are authored in the FluxML document format.

Unique characteristics of the **13CFLUX2** software suite are:

- significantly improved performance and higher numerical accuracy compared to the predecessor version 13CFLUX and other available software packages for ^{13}C-MFA;
- professional input and output via XML-based file formats and HDF5 (hierarchical data format, version 5);
- a comprehensive error handling including comprehensible error messages;
- statistical analysis of the obtained reaction rate distribution and tools for performing optimal experimental design;

- support for multi-core CPUs and computer clusters.

The following license terms apply to the software's executable files as well as to the included manuals and data files (in this agreement referred to as the "SOFTWARE"). Likewise, these terms apply to any **13CFLUX2** software update provided by Forschungszentrum Jülich GmbH, unless separate license terms accompany those items. If so, those license terms apply.

BY INSTALLING AND/ OR USING THE SOFTWARE, YOU ACCEPT THE TERMS OF THIS LICENSE AGREEMENT. IF YOU DO NOT ACCEPT IT, DO NOT INSTALL AND USE THE SOFTWARE. IF
YOU COMPLY WITH THESE LICENSE TERMS, YOU HAVE THE RIGHTS AS SPECIFIED BELOW.

2. Conditions

2.1. PUBLISHER hereby grants to LICENSEE:
a) a non-exclusive and non-transferable right to use SOFTWARE only for non-commercial academic and non-commercial scientific research purposes in its own premises and in the framework of its own research programme,
b) a non-exclusive and non-transferable right to use the SOFTWARE only for non-commercial teaching purposes.

2.2. SOFTWARE is provided to LICENSEE "as is", i.e. without any expressed or implied warranties, including, but not limited to, the warranties of merchantability and fitness for a particular purpose. In no event shall PUBLISHER or contributors be liable for any direct, indirect, incidental, special, exemplary, or consequential damage (including, but not limited to, procurement of substitute goods or services, loss of use, data or profits, or business interruption) caused and on any theory of liability, whether in contact, strict liability, or tort (including negligence or otherwise) arising in any way out of the use of SOFTWARE, even if advised of the possibility of such damage. This does not apply to damages caused by intent. PUBLISHER shall not be liable for legal or actual defects in SOFTWARE and gives no warranty. In particular, PUBLISHER accepts no liability for the correctness of the results obtained through the use of this SOFTWARE, nor shall the PUBLISHER be made liable to a third party as a consequence of the use of this SOFTWARE by LICENSEE.

2.3. PUBLISHER is not committed to deliver updates and/ or bugfix releases for the SOFTWARE. In case of optional provision of updates and/ or bugfix releases by PUBLISHER, PUBLISHER is exempted from liability for any damages caused hereby.

2.4. PUBLISHER is not committed to continual provision of SOFTWARE.

2.5. For the avoidance of doubts, no commercial use of SOFTWARE is allowed without the prior written consent of PUBLISHER.

2.6. LICENSEE is not entitled to sell SOFTWARE to third parties or to provide it to any third party free of charge without the prior written consent of PUBLISHER.

2.7. In the case of publishing or dissemination as well as oral presentation of results which refer to SOFTWARE or which contain results obtained by the use of SOFTWARE, PUBLISHER has to be mentioned as the owner and developer of the SOFTWARE as well as the internet site http://www.13cflux.net.

2.8. LICENSEE shall inform PUBLISHER in writing about any errors and deficiencies detected.

2.9. For the avoidance of doubts, any use exceeding the above mentioned scope, in particular, but not limited to, within the framework of commercial services rendered to or cooperation entered into with third parties, are subject to a separate agreement.

3. Costs

SOFTWARE is provided free of charge. Cost for shipping, handling and installation (e.g. travel) if applicable, are borne by LICENSEE.

4. Documents and Submission of Documents

4.1. The intellectual rights on documents created by LICENSEE using SOFTWARE, source code and the derived binary forms if software extensions developed by LICENSEE by using the development environment of SOFTWARE remain with LICENSEE.

4.2. IN ORDER TO ENSURE AN EXCLUSIVE NON-COMMERCIAL USE OF "SOFTWARE", IT IS NECESSARY TO SUBMIT RELATED INPUT DOCUMENTS (CONTAINING METABOLIC NETWORK SPECIFICATIONS AND MEASUREMENT DATA) TO "PUBLISHER" FOR THE PURPOSE OF STORING AND MAYBE INSPECTING THE CONTENTS. IN THIS CASE "PUBLISHER" HAS NO RIGHT TO USE THE CONTENTS OF THE SUBMITTED DOCUMENTS (AS WHOLE OR IN PART) FOR PUBLICATION OR ANY COMMERCIAL PURPOSE. FURTHERMORE, "PUBLISHER" SHALL ENSURE THAT ALL SUBMITTED DOCUMENTS ARE TREATED CONFIDENTIALLY. IN PARTICULAR, THIS MEANS THAT "PUBLISHER" HAS TO TAKE CARE THAT SUBMITTED DOCUMENTS ARE FILED IN A NON-PUBLIC AND TECHNICALLY SECURED LOCATION WHICH MAY ONLY BE INSPECTED BY TWO SELECTED EMPLOYEES OF "PUBLISHER".

4.3. As an integral part of SOFTWARE, PUBLISHER provides an automated mechanism for submission, inspection, and storage of the requested documents. SOFTWARE requires using this mechanism as a prerequisite of operation.

5. Alterations of SOFTWARE

LICENSEE is not entitled to alter or reverse engineer SOFTWARE in any way. The right to alteration of the SOFTWARE remains with PUBLISHER.

6. Alterations of this License Agreement

Any modifications and amendments to this agreement shall be made in writing. Should any provision of this agreement be ineffective or void, this shall not affect the validity of the other contractual provisions. Such invalid provision should rather be replaced by a valid provision as close as possible to the invalid provision.

7. Transferability of Rights and Obligations

Rights and obligations arising out of this agreement can be transferred to third parties only after written consent by the other party.

8. Terms of Agreement

This agreement shall come into force upon execution by both parties until 31st December 2012. Thereafter it shall automatically renew for another two years if not terminated by at least one of the parties with six month notice. Termination notice must be given in writing or by using the electronically provided procedure for termination. In case of misconduct and/ or failure to comply with any terms of this agreement PUBLISHER can immediately terminate the agreement without further notice. In the event of termination of this agreement LICENSEE is obliged to remove the programs from all data processing systems installed. If not agreed otherwise between the parties, LICENSEE shall comply with this obligation by deleting SOFTWARE from all data storage media.

9. Jurisdiction

This agreement shall be governed by the law of the Federal Republic of Germany. The Parties will settle all differences in connection with this agreement including its legal validity and effectiveness after termination on a partnership basis. If any disputes in connection with this agreement or its validity cannot be settled on a partnership basis, they shall be finally settled pursuant to the rules of the German Arbitration Institution (DIS -Deutsche Institution für Schiedsgerichtsbarkeit e.V.) by a court of arbitration, any recourse to courts of law being excluded. The place of the arbitration proceedings shall be Jülich. The language of the arbitration proceedings shall be German. The jurisdictions of the courts of law shall not be affected for measures of temporary relief. The place of jurisdiction shall be the administrative headquarters of PUBLISHER.

Forschungszentrum Jülich GmbH
Jülich, Germany,

________________ ____________________ __________________________________

Dr. D. Jürgens	Prof. Dr. W. Wiechert
Technology Transfer	IBG-1: Biotechnology

Forschungszentrum Jülich
